# The known, unknown, and the intriguing about members of a critically endangered traditional medicinal plant genus *Aconitum*

**DOI:** 10.3389/fpls.2023.1139215

**Published:** 2023-07-28

**Authors:** Richa Ashok Kakkar, Mariam Azeezuddin Haneen, Akash Chandra Parida, Gaurav Sharma

**Affiliations:** ^1^ Department of Biotechnology, Indian Institute of Technology Hyderabad, Sangareddy, Telangana, India; ^2^ Institute of Bioinformatics and Applied Biotechnology, Bengaluru, India

**Keywords:** medicinal plant, secondary metabolites, taxonomy, herbal medicine, conservation, chloroplast, transcriptome, pharmacology

## Abstract

Humanity will always be indebted to plants. In the ongoing scientific era, the ‘Herbal Revolution’ has helped discover several valuable medicinal plants and associated novel secondary metabolites from the diverse unexplored ecosystems, treating several diseases via phytotherapy. The *Aconitum* genus comprises several economically-important poisonous mountainous medicinal plant species whose unique biodiversity is on the verge of extinction due to illegal human intervention triggered habitat loss, over-harvesting, and unrestricted trading. Owing to its vast diversity of diterpene alkaloids, most species are extensively used to treat several ailments in rural parts of the world. Irrespective of this, many unexplored and intriguing prospects exist to understand and utilize this critical plant for human benefit. This systematic review tries to fill this gap by compiling information from the sporadically available literature known for ~300 *Aconitum* spp. regarding its nomenclature and classification, endangerment, plant morphology, ploidy, secondary metabolites, drug pharmacokinetics, conservation, and omics-based computational studies. We also depicted the disparity in the studied model organisms for this diverse genus. The absence of genomic/metagenomic data is becoming a limiting factor in understanding its plant physiology, metabolic pathways, and plant-microbes interactions, and therefore must be promoted. Additionally, government support and public participation are crucial in establishing conservation protocols to save this plant from endangerment.

## Introduction

Medicinal plants can be defined as those verified plants which have a history of traditional knowledge, and modern science has identified at least a few known ingredients (secondary metabolites) showing medicinal values. Since immemorial, these plants have been essential in therapeutics, especially in rural areas. The World Health Organization (WHO) has pointed out that a whopping 80 percent of the global population probably relies on traditional medicines (World Health Organisation, 2022). Most developing countries use them as herbal remedies and primary healthcare (Petrovska, 2012). However, most of their medicinal properties are not benchmarked; therefore, providing reliable evidence of their practices and products is a significant challenge for researchers. To build a solid foundation on conventional medicine practices/products and ensure all global citizens have access to safe and effective treatment, WHO and the Government of India plan to establish a dedicated institute named WHO Global Centre for Traditional Medicine in Jamnagar, Gujarat, India.

Secondary metabolites from medicinal plants can be an important source of potential drug leads. Many commonly used drugs have their origin from plant sources; for example, Salicin, a natural substance obtained from the bark of the willow tree *Salix alba L*., is the source of aspirin; Morphine, the first commercially important drug used since 1803, is obtained from *Papaver somniferum L.* (opium poppy) (Dias et al., 2012); Artemisinin drug from *Artemisia annua*; ephedrine, a central nervous system (CNS) stimulant, from *Ephedra sinica*; Reserpine, an antihypertensive agent, isolated from *Rauwolfia serpentina* used for the snakebite treatment; vinca alkaloids (vinblastine and vincristine), well-known for their usage in chemotherapy (as an antimicrotubule agent), isolated from *Catharanthus roseus* (Madagascar periwinkle); to name a few (Cragg and Newman, 2013). Along with their medicinal usage owing to their drug molecules, they are also a good source of nourishment, condiments, and oil, which can be directly or indirectly related to human/animal health (Pandey et al., 2013).

**Figure 1:**
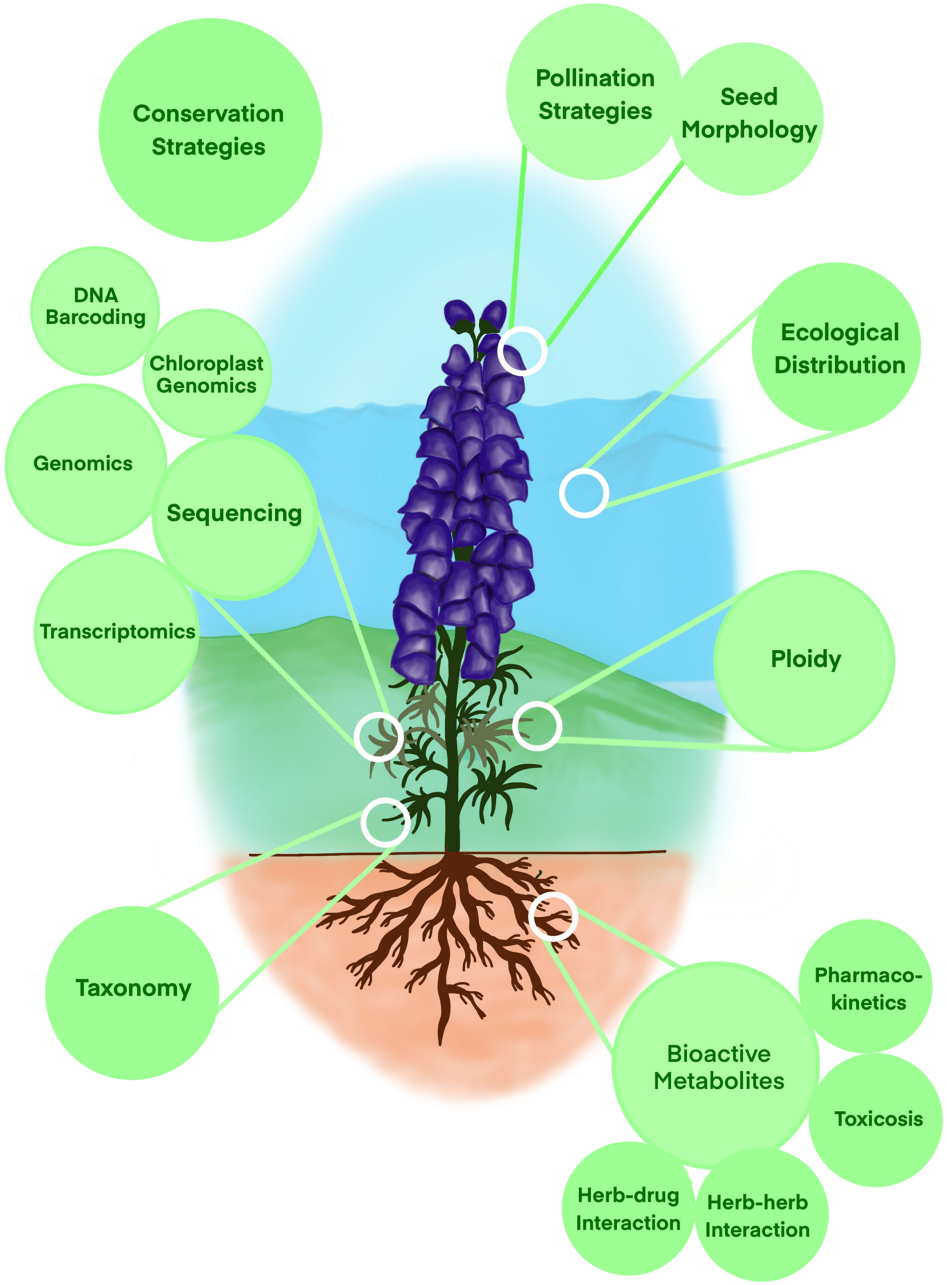
Cartoon depiction representing various research fields reviewed here to understand *Aconitum* diversity, physiology, taxonomy, metabolites, and pharmacokinetics.

India is well-known for its three diversified traditional medicinal systems—Ayurveda, Siddha, and Unani (Pandey et al., 2013; Mohanraj et al., 2018). The disparate terrain throughout the country, ranging from the Himalayas to the plains and from the ocean to the desert, helps in the diversity, origin, survival, and dispersion of numerous medicinal plants. India’s immense wealth of medicinal plants can be attributed to 9,500 species having ethnobotanical importance and 7,500 species being used in indigenous health practices and modern medicine (Sharma and Pandey, 2013). However, because of unsustainable usage for general use and export, unwarranted utilization, overexploitation, and unskilled harvesting, >90% of these valuable medicinal plants face a threat, and many are on the verge of extinction. This review is focused on one such critically endangered traditional Indian medicinal plant, belonging to the genus *Aconitum*, sporadically present in the Middle Himalayas ranges. *Aconitum* plants belong to the family Ranunculaceae within the clade Angiosperms and are known via several common names throughout the world such as aconite, wolf’s-bane, devil’s helmet, monkshood, women’s bane, or queen of poisons. These herbaceous perennial plants have around 300 representative species (**Table S1**), scattered sparsely in the moisture-retentive well-draining soils of the mountainous ranges within North America, Europe, and Asia continents. This review focuses on all research aspects studied in the past and compiles all such information in a systematic manner (**Figure 1**). In the end, we also discussed several research areas, especially in terms of plant conservation and omics-based functional understanding where more attention is expected from the scientific community.

## History of the word ‘*Aconitum*’

The word “*Aconitum*” originated from the word “Akonitos” which means “without struggle” which probably refers to the manner of death caused by its poison. The name also has an obscure etymology: “ak” means sharp or pointed, and “konos” means cone referring to the plant’s pointed leaves. There are several instances in Greek mythology concerning the plant getting its name from a Greek city called Acona where it was first discovered - the Hill of Aconitus, where Hercules fought a three-headed dog called Cerebrus. It is said that the saliva of the Cerebrus rendered *Aconitum* poisonous forever. The Roman Emperor Claudius was overthrown after being poisoned with it by Nero. The Roman writer Ovid mentions *Aconitum* as a stepmother’s poison in his works. In Shakespeare’s Romeo and Juliet also, the lead characters commit suicide after consuming this very poison. *Aconitum* has been nicknamed ‘wolf’s bane’ or ‘arrow poison’ as arrows dipped in it were used to kill wolves (El-Shazly et al., 2016; Vogel, 2018).

## Classification, taxonomy, and evolution of *Aconitum* spp.

The *Aconitum* genus belongs to the buttercup family, or Ranunculaceae, representing herbaceous annuals or perennial members widely present amidst the alpine ecosystems in the Northern Hemisphere. This family comprises more than 2300 species globally, with the highest diversity in the northern extratropical zone (The Plant List, 2010). Multiple lineages in this family diverged as early as 125.8– 123.0 Mya according to the “accelerated angiosperm evolution” hypothesis (Wang et al., 2016). The split between *Aconitum* and other genera occurred around 24.7 Mya (Park et al., 2020), and the separation between *Aconitum* L. subgen. *Aconitum* and subgen. *Lycoctonum* (DC.) Peterm. has been estimated to be approx. 8.24- 20.7 Mya (Wang L. et al., 2009). As per the *Aconitum* pollen deposits recovered from Central Europe, the occurrence of *Aconitum* can be traced back to the Late Miocene, thereby accentuating the period’s significance in the development of European mountain and high-mountain flora. Temperate forests that once covered the southern shores of Central and Eastern Parathetys (Western Asia) were gradually replaced by open woodlands throughout the Late Miocene. Forest fragmentation, the emergence of open spaces, the growth of grasslands and xerophytic plant groups, and the loss of subtropical species from the ancient flora would have added to *Aconitum* isolation and evolutionary divergence (Nakai, 1953).

Over the last twenty years, several family Ranunculaceae phylogenetic studies have been performed based on diverse characteristics such as morphology, restriction site mapping, nuclear sequences, and chloroplast sequences (Johansson, 1998; Wang W. et al., 2009; Emadzade et al., 2010). Due to their varying morphology, the taxonomic classification of *Aconitum* has been complicated, leading to several changes leading up to the current classification model. *Aconitum* was first classified as a subgenus in 1824 by de Candolle into three sections: Anthora, Napellus, and Cammarum (Candolle, 1857). It was re-classified by Nakai in 1953, wherein *Aconitum* was made a separate genus with three subgenus- *Asianthora, Napellus*, and *Cammarum* (Nakai, 1953). This classification was deemed unreliable when several differences were found in inflorescences, branching of stem, hairiness, and dentation of stamens within the population. Then in 1979, W. T. Wang classified it based on the shape of sepals and petals, the shape and hairiness of stamen filaments, and the carpel number into two sections - Sinaconitum and Aconitum (Luo et al., 2005; Xiao et al., 2006). To improve it further, in 1995, Tamura classified the genus based on inflorescence, the number and arrangement of daughter tubers, the shape of seeds, the structure of the embryo sac, and the presence or absence of petioles of cauline leaves (Tamura, 1995). He divided it into five sections - Helleboroideae, Ranunculoideae, Isopyroideae, Thalictroideae, and Hydrastidoideae. At that time, one of the significant concerns in classifying tribes in the Ranunculaceae family was their floral evolution, as substantial variation was observed in the perianth between tribes. Following these criteria, W. T. Wang classified the Ranunculaceae family into five subfamilies (Wang W. et al., 2009) – Coptidoideae, Glaucidioideae, Hydrastidoideae, Ranunculoideae, and Thalictroideae. The Ranunculoideae subfamily comprises ten tribes: Adonideae, Anemoneae, Asteropyreae, Callianthemeae, Caltheae, Cimicifugeae, Delphinieae (having genus *Aconitum*), Helleboreae, Nigelleae, and Ranunculeae. This classification is the most followed one to date. Recently it has also been proposed that a flat petal with a short claw existed in the ancestors of the Ranunculaceae family, and later it evolved into various elaborate forms as present today (Delpeuch et al., 2022), further supporting W. T. Wang’s classification using floral evolution.

According to W. T. Wang’s classification, *Aconitum* belongs to the tribe Delphinieae and has been further classified into two subgenera, *Lycoctonum* and *Aconitum* (**Table S2**). The subgenus *Aconitum* includes around 300 species further divided into two sections: Sinaconitum and Aconitum. Sinaconitum has only *A. polycarpum*, whereas section Aconitum consists of the remaining species further divided into eleven series named Volubilia, Volubilia (Inflata), Grandituberosa, Racemulosa, Rotundifolia, Brachypoda, Ambigua, Stylosa, Bullatifolia, Brunnea, and Tangutica (Xiao et al., 2006). Similarly, subgenus *Lycoctonum* has only one section named the same, which is further divided into five series known as Lycoctonia, Scaposa, Volubilia, Longicassidata, and Crassiflora. Based on several different non-molecular characteristics, such as phytochemical, cytological, anatomical, and palynological (study of plant pollen and spores) characteristics, *Aconitum gymnandrum* has been removed from the genus *Aconitum* and converted into a separate genus *Gymnaconitum*, with only one species, i.e., *Gymnaconitum gymnandrum Maxim* (Wang et al., 2013).

Based on toxicity scales too, the genus *Aconitum* has been classified into Dula and non-Dula. *A. bulleyanum Diels, A. delavayi Franch., A. stapfianum Hand.-Mazz., A. episcopale Levl., A. vilmorinianum Kom.*, and *A. contorium Finet et Gagnep* have been classified as Dula as they are used to counteract the toxicity of other *Aconitum* plants. Chih Wu Shih Khao first mentioned Dula in his book Wu Chi Chun and reports that the original plant belonging to Dula is *A. contortum* (Qinger, 1990). They do not contain the alkaloids aconitine, hypaconitine, and mesaconitine.

It must be mentioned that morphological and molecular markers for identifying *Aconitum* spp. are minimal due to morphological similarity among species. Moreover, genome information is also unavailable, which could have been vital for understanding and classifying these species. Based on such close similarities and minute variations, there is an immediate need for a more resolved phylogenetic framework.

## Distribution and endangerment of *Aconitum* spp.

Around 300 *Aconitum* spp. (**Table S1**) are present all over the world, reigning majorly amidst the cold temperate regions of the northern hemisphere predominantly located in the mountain meadows of East and South-eastern Asia and Central Europe, to a lesser extent in North temperate mountainous regions and a meager subset of species in Western North America and Eastern United States (Luo et al., 2005). Europe is home to 94 taxa, of which 22 are native, and 28 are non-native species (hybrid species). Most *Aconitum* spp. are found in southwestern China in the Hengduan Mountains region, i.e., around 100 species, which are extensively used in their traditional Chinese medicinal therapy (Li, 1988). Based on their relative abundance in the Himalayan region covering India, Nepal, Bhutan, South Tibet, and Pakistan, *Aconitum* is extensively used in Asia in the local and traditional medicinal systems. In India, 27 *Aconitum* spp. expand to the alpine and subalpine regions of the Himalayas. *Aconitum* tubers hold most of the diterpenoid alkaloids such as Aconitine, Mesaconitine, and Hypaconitine, which upon processing with heat or alkaline treatment, break down to form Benzoylaconine, Aconine, and Pyroaconine. These processed tubers have a very high therapeutic index, thereby better for medicinal uses (El-Shazly et al., 2016). Due to the high therapeutic index of their secondary metabolites, the demand for the plant is more than its production, and hence, *Aconitum* has been deemed endangered by the International Union for Conservation of Nature (IUCN). Based on this, Red Data Book has classified several *Aconitum* spp. under categories of endangered, critically endangered, vulnerable, near threatened, and of least concern. According to the red data book, *A. chasmanthum*, *A. infectum Greene* (Treher, 2015), and *A. heterophyllum* have been termed critically endangered*; A. lasiocarpum*, *A. nagarum Stapf* (Nayar and Sastry, 1987), and *A. noveboracense* (Treher, 2022) are near threatened*; A. napellus, A. degenii*, and *A. coreanum* are of ‘least concern’; *A. violaceum, A. deinorrhizom Stapf*, and *A. falconeri Stapf* var. *latilobum Stapf* (G.B. Pant National Institute of Himalayan Environment and Sustainable Development, 1992) are vulnerable. Of all these, *A. balfourii* is highly endangered due to the presence of pseudaconitine in its tubers, an extremely toxic alkaloid with incredible therapeutic benefits.

This genus comprises 24-27 species in India, including *A. chasmanthum Stapf ex Holmes, A. violaceum Jacq. ex Stapf, A. heterophyllum Wall., A. ferox Wall ex Ser., A. deinorrhizum Stapf., A. balfourii Stapf*, etc., mainly distributed in subtropical, alpine, and subalpine regions of Himalaya (Botanical Survey of India, 2014). The existence of around 16 species has been reported to be critical (Ali et al., 2019). Within India, Jammu and Kashmir have the richest diversity of medicinal plants in general because of the ranges of habitats the state provides - Kashmir valley belongs to the temperate region, Jammu represents the sub-tropical and tropical region, and in contrast, Ladakh is a cold desert region (Gairola et al., 2014). Around 50% of the medicinal plants mentioned in the British Pharmacopoeia are growing in Jammu and Kashmir (Rasool et al., 2016). Along with Jammu and Kashmir, several *Aconitum* spp. has been reported from other Himalayan ranges covering states such as Himachal Pradesh, Sikkim, and Uttaranchal (Kala et al., 2006). Recently, a new *Aconitum* sp., *A. sikkimensis*, has been discovered in Sikkim (eastern Himalayas of India), which has a similar shape and flower color as *A. hookeri* but differs in plant height, leaf shape, degree of lamina dissection, number of bracts and their shape and size and length of petiole. It has also been deemed “Critically Endangered” by IUCN (Singh et al., 2021).

Some *Aconitum* spp. from the Himalayas are as follows:

- ***Aconitum heterophyllum*** is an herbaceous, perennial, rhizomatous plant known as aconite, monkshood, devil’s helmet, and blue rocket, widely distributed in the temperate parts of the Western Himalayas. *A. heterophyllum* is mainly found in the alpine and sub-alpine regions such as Gulmarg, Khilanmarg, Sonamarg, etc. These regions are at an elevation of around 3000-3500m above sea level. It is mainly distributed in regions with loose soil, fewer pebbles, and moist, open alpine slopes.
- A variety called ***Aconitum heterophyllum* var. *bracteatum***, is now known as a separate species called ***A. kashmiricum Stapf ex Coventry*** and locally known as ‘Pevak’. This variety is considered an adulterant of *A. heterophyllum* and a critically endangered medicinal herb endemic to the Himalayas region in Kashmir (Stapf, 1905; Chaudhary and Rao, 1998; Jabeen et al., 2013).
- ***Aconitum napellus*** is found in the temperate, alpine Himalayas with altitudes ranging from 3000m to the highest point of vegetation. The dried roots have a conical or tapering shape and appear larger with a knotty crown. The stem’s exquisite raceme of dark blue helmet-shaped blooms is crowned by the uppermost leaves, which are simpler than the lower leaves and gradually transition into bracts. It is known for its antidiabetic activity (Aconitum napellus, Linn, 2018; Shoaib et al., 2020).
- ***Aconitum chasmanthum Stapf ex Holmes*** grows at high altitudes in Kashmir. Its roots resemble *A. napellus*, a native and endemic to western and central Europe, leading to its frequent wrong identification (Chakravarty and Chakravarti, 1954). However, in comparison to the European *A. napellus*, Indian *A. chasmanthum* is seven times more potent in its alkaloid content, which is the reason for its great demand in the world market (Ali et al., 2019).
- ***Aconitum laeve* Royle** is native to the Northwest Himalaya, distributed mainly from Chitral to Kumaon at an altitude of 2500-3500m, mostly in the forest areas of Jammu and Kashmir, Himachal Pradesh, Uttar Pradesh, Pakistan, and Nepal.
- ***Aconitum violaceum Jacquem*** is mainly distributed over the alpine slopes of the Himalayan region at an altitude of 3500-4000m in India, Pakistan, Nepal, Jammu and Kashmir, Himachal Pradesh, and Uttar Pradesh. They have been listed within the vulnerable category by IUCN.
- ***Aconitum balfourii Stapf*** is endemic to the alpine and subalpine belts of the Indian Himalayan region, most widespread in Kumaon and Garhwal Himalayas on shady slopes from 3000 to 4200 m.

In Pakistan, this genus is represented by 11 species (*A. laeve, A. moschatum, A. heterophyllum, A. ovatum, A. kashmiricum, A. deinorrhizum, A. violaceum, A. Chasmanthum, A. rotundifolium, A. soongoricum*, and *A. curvipilum* (Rledl, 1993) and seven varieties amidst which the following species are under severe threat due to habitat loss and excessive harvesting - A*. chasmanthum Stapf ex Holmes* being critically endangered, *A. heterophyllum Wall. ex Royle* is endangered, and *A. violaceum Jacquem. ex Stapf* in a vulnerable state (Alamgeer et al., 2018).

In China, *Aconitum*’s center of diversity and speciation is in the southwest region, namely in the Hengduan Mountains region where 166 out of 211 identified species are indigenous (Flora of China, 2008). The South-Western Provinces of Ytinnan and Tibet are even more abundantly endowed with species than the North-East Provinces (Manchuria) and Szechuan, where the very broken landscape of high mountain ranges and deep, narrow valleys provide suitable conditions for growth. *A. carmichaelii, A. nagarum, A. ouvrardianum, A. stylosum*, and *A. episcopale* are the well-known species used as arrow poison.

## Morphology of *Aconitum* spp.

**Figure 2:**
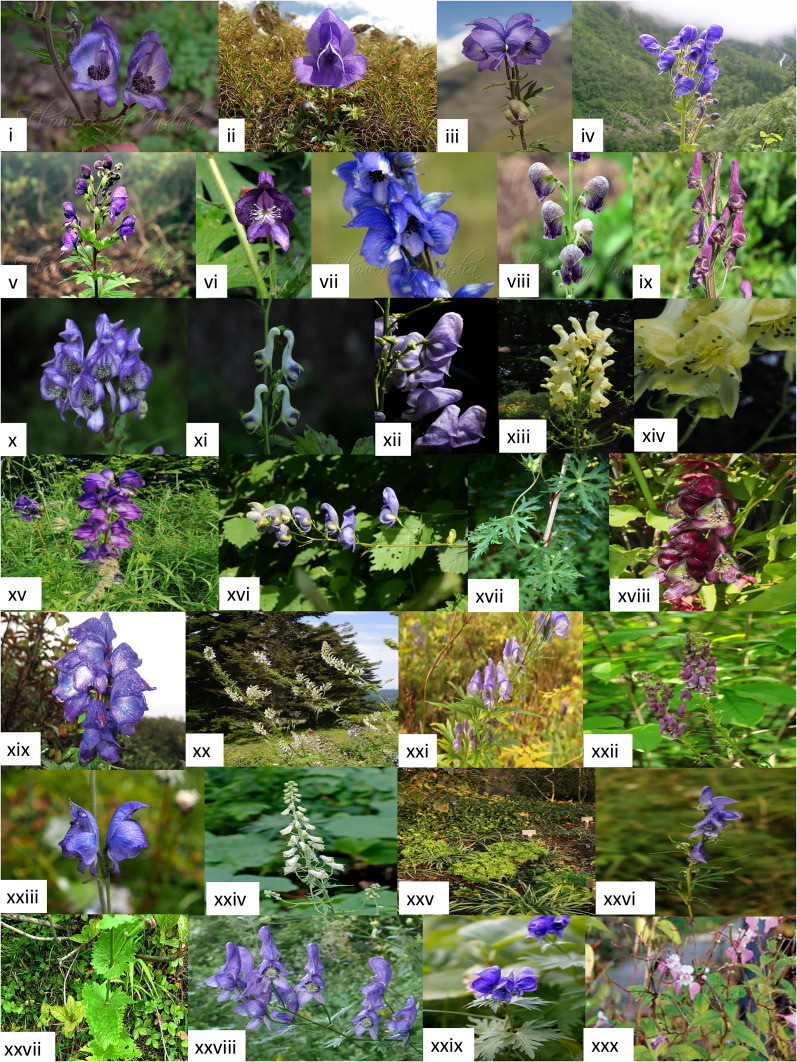
Flower and plant morphology images of several *Aconitum* spp. compiled from diverse, authentic web pages as mentioned below: i. *A. heterophylloides* (Nepal Monkshood) - https://www.flowersofindia.net/catalog/slides/Nepal%20Monkshood.html,ii. *A. fletcheranum* (Fletcher’s Monkshood) - https://www.flowersofindia.net/catalog/slides/Fletcher%27s%20Monkshood.html, iii. *A. violaceum* (Violet Monkshood)- https://powo.science.kew.org/taxon/urn:lsid:ipni.org:names:707944-1, iv. *A. heterophyllum* var bracteatum (Himalayan Monkshood) - http://www.flowersofindia.net/catalog/slides/Himalayan%20Monkshood.html, v. *A. lethale* (Balfour’s Monkshood) - https://www.flowersofindia.net/catalog/slides/Balfour%27s%20Monkshood.html, vi. *A. laeve* (Grape-leaved Monkshood) - https://www.flowersofindia.net/catalog/slides/Grape-Leaved%20Monkshood.html, vii. *A. chasmanthum* (Gaping Monkshood) - https://www.flowersofindia.net/catalog/slides/Gaping%20Monkshood.html, viii. *A. heterophyllum* (Greenish Himalayan Monkshood) - https://www.flowersofindia.net/catalog/slides/Greenish%20Himalayan%20Monkshood.html, ix. *A. hopeiense* - https://powo.science.kew.org/taxon/urn:lsid:ipni.org:names:326013-2, x. *A. kusnezoffii* - https://powo.science.kew.org/taxon/urn:lsid:ipni.org:names:707505-1, xi. *A. longecassidatum* - https://duocet.ibiodiversity.net/index.php?title=%E6%96%87%E4%BB%B6:Aconitum_longecassidatum_01.jpg, xii. *A. carmichaelii* - https://powo.science.kew.org/taxon/urn:lsid:ipni.org:names:707241-1, xiii. *A. lamerckii* - https://powo.science.kew.org/taxon/urn:lsid:ipni.org:names:707513-1, xiv. *A. lycoctonum* - https://powo.science.kew.org/taxon/urn:lsid:ipni.org:names:707564-1, xv. *A. napellus* - https://powo.science.kew.org/taxon/urn:lsid:ipni.org:names:326013-2, xvi. *A. nasutum* - https://powo.science.kew.org/taxon/urn:lsid:ipni.org:names:326013-2, xvii. *A. episcopale* - https://powo.science.kew.org/taxon/urn:lsid:ipni.org:names:707335-1, xviii. *A. hemsleyanum* - https://powo.science.kew.org/taxon/urn:lsid:ipni.org:names:707423-1, xix. *A. spicatum* – https://www.researchgate.net/figure/Aconitum-spicatum_fig2_283965239, xx. *A. orientale* – https://powo.science.kew.org/taxon/urn:lsid:ipni.org:names:707662-1, xxi. *A. sachalinense* - https://powo.science.kew.org/taxon/urn:lsid:ipni.org:names:707782-1, xxii. *A. sinomontanum* - https://powo.science.kew.org/taxon/urn:lsid:ipni.org:names:707823-1/images, xxiii. *A. tanguticum* - https://powo.science.kew.org/taxon/urn:lsid:ipni.org:names:707870-1, xxiv. *A. vulparia* - https://powo.science.kew.org/taxon/urn:lsid:ipni.org:names:707955-1/images, xxv. *A. cammarum* - https://powo.science.kew.org/taxon/urn:lsid:ipni.org:names:707233-1/images, xxvi. *A. jeholense* var. angustius - https://powo.science.kew.org/taxon/urn:lsid:ipni.org:names:951995-1/images, xxvii. *A. heterophyllum* (Greenish Himalayan Monkshood) - https://www.flowersofindia.net/catalog/slides/Greenish%20Himalayan%20Monkshood.html, xxviii. *A. variegatum* - https://en.wikipedia.org/wiki/Aconitum, xxix. *A. uncinatum* - https://plants.ces.ncsu.edu/plants/aconitum-uncinatum/, xxx. *A. balfourii* - https://vikaspedia.in/agriculture/crop-production/package-of-practices/medicinal-and-aromatic-plants/aconitum-balfourii.

All *Aconitum* spp. are perennial herbs with tuberous roots and 0.91- 1.21m in height. It grows best in well-drained soils. Its leaves are directly attached to the stem, not via a petiole, making them cauline. Each leaf has a lobed leaf blade of 3-7 segments with toothed margins known as dentate (Ali et al., 2019), except in *A. chasmanthum*, which has no dentate pattern. Its bilaterally symmetrical flowers appear purple, dark blue, or white. Each flower has six sepals, of which one sepal at the top of all other sepals is beaked (Raina et al., 2011), giving the flower a hooded appearance from which it has earned its nickname “monkshood” (Brink, 1982; Furbee, 2009; Flora of North America, 2016) (**Figure 2**). *Aconitum* spp. also have characteristic fusiform tuberous root morphology, and its daughter tubers are used for propagation. These tubers are known to have significantly higher aconitine (its major secondary metabolite) content than other parts, i.e., stem, leaves, buds, etc., irrespective of their growth at any altitude (Rawat et al., 2014). *A. columbianum* has parent and daughter tubers placed adjacent, whereas *A. uncinatum* has tubers separated by elongated rhizomes (Brink, 1982). For most of these plants, their flowering season is between September and October, with 20 days of peak flowering. The life cycle of *Aconitum* spp. starts with a parent tuber, producing the future flowering stems and leaves in the spring season. Simultaneously the daughter tuber also starts growing and keep growing all through the summer and fall. The parent tuber eventually dies at the end of the year once the seeds have ripened. As a result, the daughter tuber takes over the parent tuber’s function the following spring (Bisset, 1981). Reproductive biology knowledge is crucial for conserving, managing, and recovering threatened species. Despite *Aconitum*’s wide range of medicinal uses, research on its floral biology and breeding habits has not been explored extensively (Nautiyal et al., 2009).

## Pollination strategies and benefits of nectar secretion in *Aconitum* spp.

Plant pollination by insects is a well-known phenomenon. Additionally, any pollen that the insects ingest is infertile and cannot fertilize other flowers. The flowers of the dichogamous *Aconitum* have a male and female phase (Nautiyal et al., 2009). The primary pollinators of *Aconitum* are long-tongued bumblebees (*Bombus hortorum* and *B. pascuorum*), whereas honeybees and short-tongued bumblebees (*Bombus terrestris* and *B. lucorum*) are mostly thought of as nectar robbers (Jacquemart et al., 2019). To maximize pollen transport to flowers in the female phase, the male phase must draw pollinators and safeguard the pollen. As *Aconitum* toxicity can also harm pollinators, it is astonishing how *Aconitum* attracts pollinators. To produce pollen, the *Aconitum* flowers open with a male phase which lasts for five or six days, during which there is higher production of scents and nectar, following which the organs wither, and the female organs become exposed to pollen. Compared to the female phase, the *Aconitum* male phase produces four times as much nectar and volatile chemicals (Jacquemart et al., 2019). According to a recent study, *Polemonium caeruleum* male flowers secrete nectar rich in sucrose, demonstrating the male bias in nectar production (Ryniewicz et al., 2020).

While the nectar is attractive, the pollen is lethal, and the success of *Aconitum* pollination depends on this combination. One way to think of pollen toxicity is as a chemical defense that aids plants in reducing herbivory and over-pollination. As alkaloids can potentially repel, hurt, or even kill visitors in large doses, they may dissuade non-pollinating insects. This might minimize pollen waste and enhance pollen transport between plants. However, the visitors drink more of the less harmful nectar while collecting less pollen for larval feeding. Pollination is aided by toxic pollen on the insect bodies, thereby improving pollen transfer efficiency (Gosselin et al., 2013; Mayer et al., 2014). High male fitness, the deterrence effect of deadly pollen, and the high production of sugary nectar combine to ensure the reproductive success of this specialized protandrous plant species (Jacquemart et al., 2019).

## Seed morphology helps *Aconitum* spp. in their ecological adaptation

Seed morphology has been acknowledged as a significant source of valuable phylogenetic information due to its high consistency. The seed morphology of several angiosperm taxa has already been thoroughly examined (Oh et al., 2008; Mundhra and Roy, 2012; Ghimire et al., 2017), along with phenetic or phylogenetic analysis at the genus level. The variance in seed morphology has previously been utilized in plant systematics in various ways - character-state evolution, phylogenetic inference, and taxonomy circumscription (Ghimire et al., 2017). Macro- and micromorphological seed traits within and among the various genera serve as valuable taxonomic evidence that aids in classifying taxa (Fawzi, 2011; Patil et al., 2015). Seed coat morphology is a unique characteristic for taxonomic and evolutionary investigations as it aids in classification, establishing evolutionary links, illuminating the seed coat’s adaptive relevance, and serving as genetic markers for identifying genotypes (Verma and Kumar Avinash Bharti, 2017).

**Figure 3:**
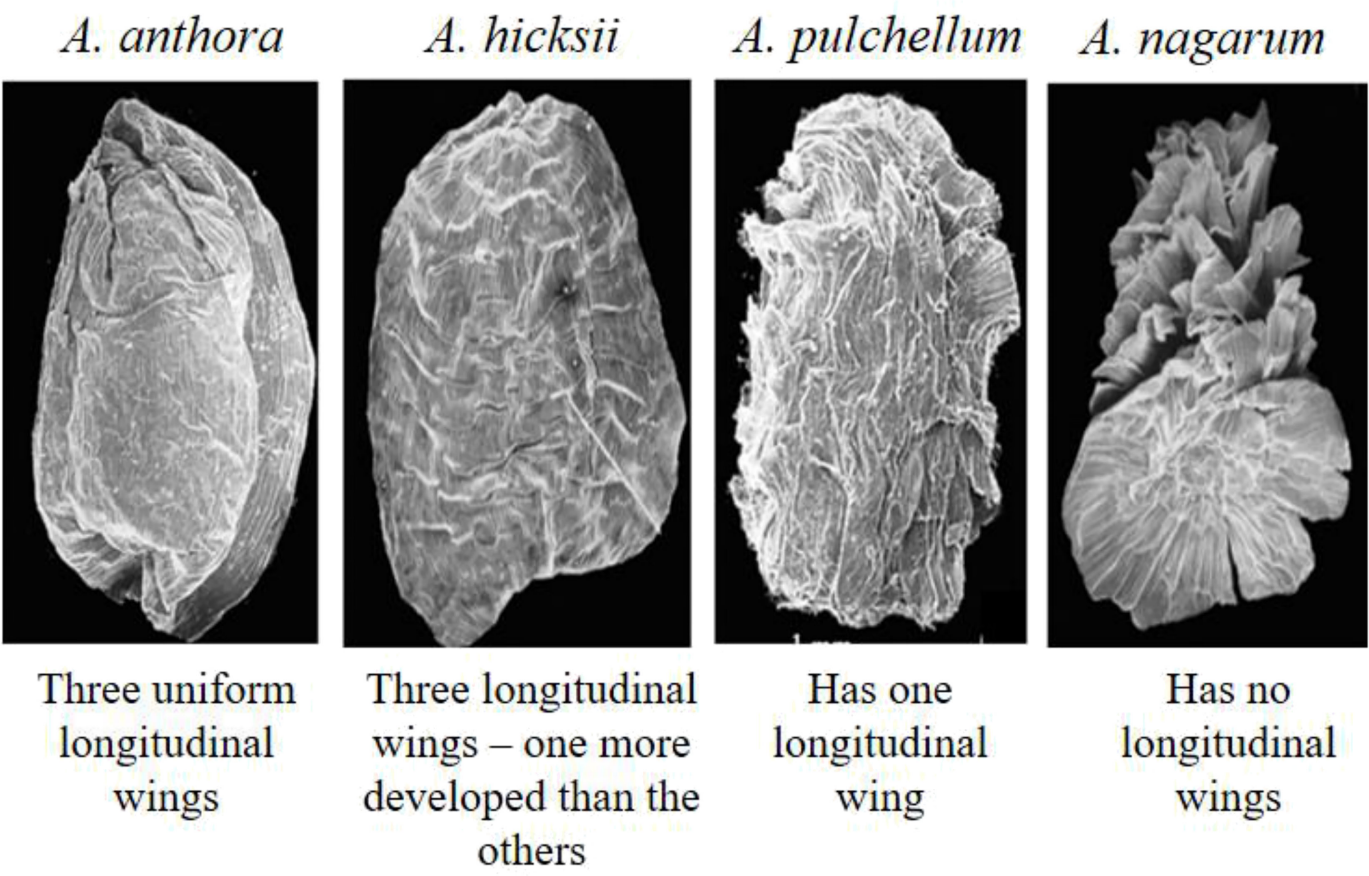
Seed morphology of Aconitum spp. (Adapted from Hui et al., 2013) – 1. A. anthora, 2. A. hicksii, 3. A. pulchellum, 4. A. nagarum.

*Aconitum* spp. and subspecies have triangular and pyramidal seeds. Each seed has three faces, and a base, with the hilum region located in its center with longitudinal wings, found on the three edges. Because of the ridges, the seed faces are either smooth or rugose. In an attempt to assess the taxonomic importance of *Aconitum*, the scanning electron microscopy (SEM) of the seed morphology of 57 species and five varieties indicated a high degree of consistency with warty ornamentation in their integument epidermal cells (Hui et al., 2013). The three subgenera of this genus, *i.e., Lycoctonum*, *Aconitum*, and *Gymnaconitum*, show distinct variances in seed gross morphology – *Gymnaconitum* has round or elliptic-shaped integument epidermal cells, whereas *Lycoctonum* and *Aconitum* have rectangular shaped epidermal cells (Balkrishna Ghimire, 2015). *A. novoluridum* seeds may be the most primitive seed form in the genus *Aconitum* since they have longitudinally very thinly winged along three ridges and are rarely squamate (Borthakur and Dahal, 2017). The seeds have been divided into four categories based on the longitudinal wings (**Figure 3**) (Cappelletti and Poldini, 1984; Hui et al., 2013)–

1. Smooth faces and three uniformly developed longitudinal wings along the edges; the simplest variety; –seen in *A. tanguticum, A. anthora.*
2. Seeds with three longitudinal wings, one of which is distinctly more developed than the other two, but no visible edges –seen in *A. hicksii*, *A. brunneum*, *A. napellus.*
3. Having one longitudinal wing with no visible edge; have transverse scaly wings from across the ribs –seen in *A. pulchellum, A. variegatum, A. paniculatum, A. angustifolium.*
4. Seeds with no longitudinal wings; a broader transverse membranous fin on the opposite side –seen in *A. nagarum, A. iochani, A. vulparia and A. lamarckii.*

*Aconitum* seed morphology is characterized by a decrease in longitudinal wings on the edges and a synchronous growth of ridges on the seed faces which extend into the wing. Developing a transverse wing on the seed is an ecological adaptation to improve the seed’s floating ability. The presence of wings overall influences seed dispersal effectiveness via floating ability and hygroscopic movements separating seeds from each other (Cappelletti and Poldini, 1984). Several species, such as *A. variegatum* and *A. vulparia* are highly mesophilous-hygrophilous. On the contrary, *A. anthora*, a xerothermic remnant species, has the simplest seed shape. However, *A. napellus*, which grows in open environments such as rocky soil (Plants For A Future, 1997), has seed morphology between *A. anthora* and *A. variegatum* or *A. vulparia*. These examples suggest that more elaborate seed coat traits could be present in other aconites providing diverse ecological adaptations to the respective plant.

## Diversity at ploidy level in *Aconitum* spp.

Polyploidy is a whole genome duplication event caused by meiotic failure leading to spontaneous non-disjunction of chromosomes and resulting in a cell that contains more than two whole sets of chromosomes. Such plants have characteristic large leaves, flowers, and fruits and are also tolerant to various environmental stresses and pathogen attacks as they can express multiple copies of the genes responsible for secondary metabolite production, serving as their defense mechanism (Kumar, 2021). Polyploidy increases the number of beneficial genes in a plant, minimizes the effect of mutated genes, and improves overall plant health (Lavania, 2020). Induction of polyploidy in medicinal plants can enhance the production of secondary metabolites used to make several therapeutic compounds such as morphine, codeine, quinine, etc. For example, the production of scopolamine used to treat motion sickness was increased to 200% in *Hyoscyamus reticulatus* after tetraploid induction (Madani et al., 2021). Improving terpene synthesis in plants through polyploidy has produced excellent outcomes - 56% higher artemisinin content was obtained from tetraploid *Artemisia annua*, which is very high compared to diploids (Lin et al., 2011). However, polyploidy can also negatively impact secondary metabolite production as the genome content exceeds the cell membrane’s surface area and leads to genetic instability. Nonetheless, it reduces the burden of harvesting wild medicinal plants (Madani et al., 2021). Overall, polyploidy is a powerful method to increase the production of alkaloids, terpenes, and polyphenols in medicinal plants, which can lead to the discovery of new drugs and boost the herbal medicine industry.

**Figure 4:**
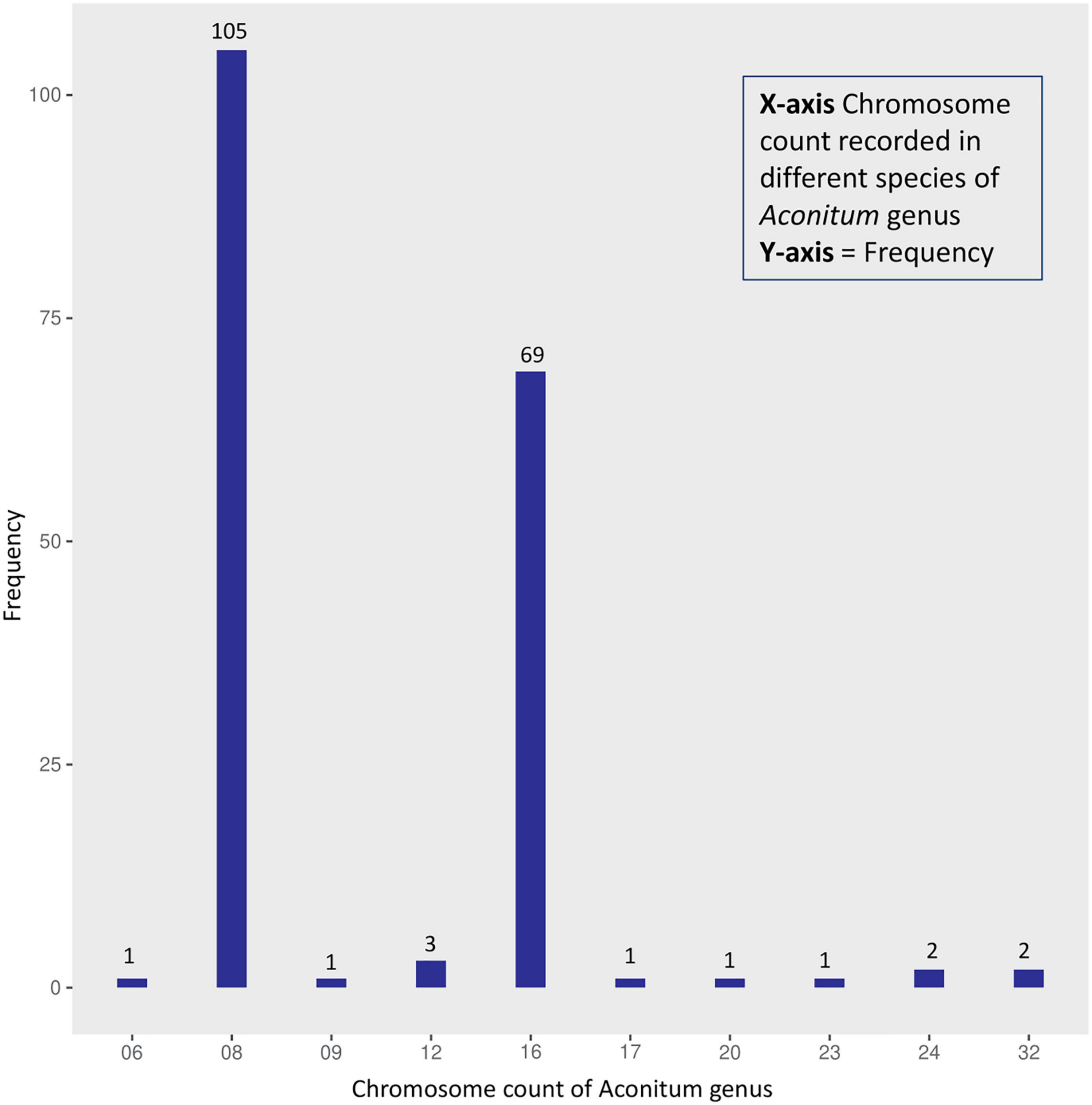
Frequency distribution of ploidy levels based on data available at the Chromosome Counts Database (CCDB) server.

Karyotype studies focused on chromosome count are essential to understand the evolution and endemicity of a species (Chung et al., 2011). Most *Aconitum* spp. exhibit bimodal karyotypes, meaning they have two sets of chromosomes of contrasting sizes (Joachimiak et al., 2018; Báez et al., 2019). Most of these plants have eight chromosomes, two of which are large and submetacentric, whereas six are small and primarily submetacentric (Joachimiak et al., 2018) (**Figure 4**). However, *A. fletcheranum* is the only *Aconitum* sp. to have six chromosomes with (2n=12), whereas others are found to be diploid with (n=8), tetraploid with (2n = 32) and with (2n = 48); *A. apetalum*, is a hexaploid exhibiting highest ploidy levels for *Aconitum* (Hong et al., 2016). Rpl32 gene, present in multiple divergent copies in *Aconitum*, underwent two significant duplication events, which were detected between 11.9 and 24.7 Mya, according to phylogenetic analyses (Park et al., 2020). A recent sequence rate analysis study has reported that eight rpl subunits have an increased evolutionary rate as opposed to those in other genera, highlighting paralogs’ role in evolution (Park et al., 2020). Such intercellular gene transfers reveal its ancient polyploidization.

Polyploidy is the driver of evolution as opposed to being a dead end. There is gene redundancy in polyploid plants because of two ancient whole genome duplication events responsible for restructuring plant genomes conferring the advantage of adaptability to plants to survive harsh climatic ordeals. Higher ploidy levels can be observed moving from the equator to the poles, suggesting the presence of polyploidy in *Aconitum* spp. The presence of *Aconitum* spp. in harsh colder climates also explains its ability to form tubers that can regenerate during favorable environmental conditions (Rice et al., 2015; Lavania, 2020).

## Secondary metabolites of *Aconitum* spp. and their impact on human health

Secondary metabolites are a crucial part of the plant defense system and have critical applications for human use in medicines, agrochemicals, pigments, food additives, and biopesticides. In addition, several naturally occurring secondary metabolites are being modified to be used as commercial drugs. Given the varied application of secondary metabolites, several methods, such as plant cell and tissue culture techniques, plant genetic transformation, transcription factor engineering, and cellular compartment targeting, have been employed to increase the production of secondary metabolites in plants. Secondary metabolites can be broadly divided into hydrophilic and hydrophobic; however, the phytochemicals of particular interest are terpenoids, phenolic compounds, alkaloids, and non-protein amino acids.

**Figure 5:**
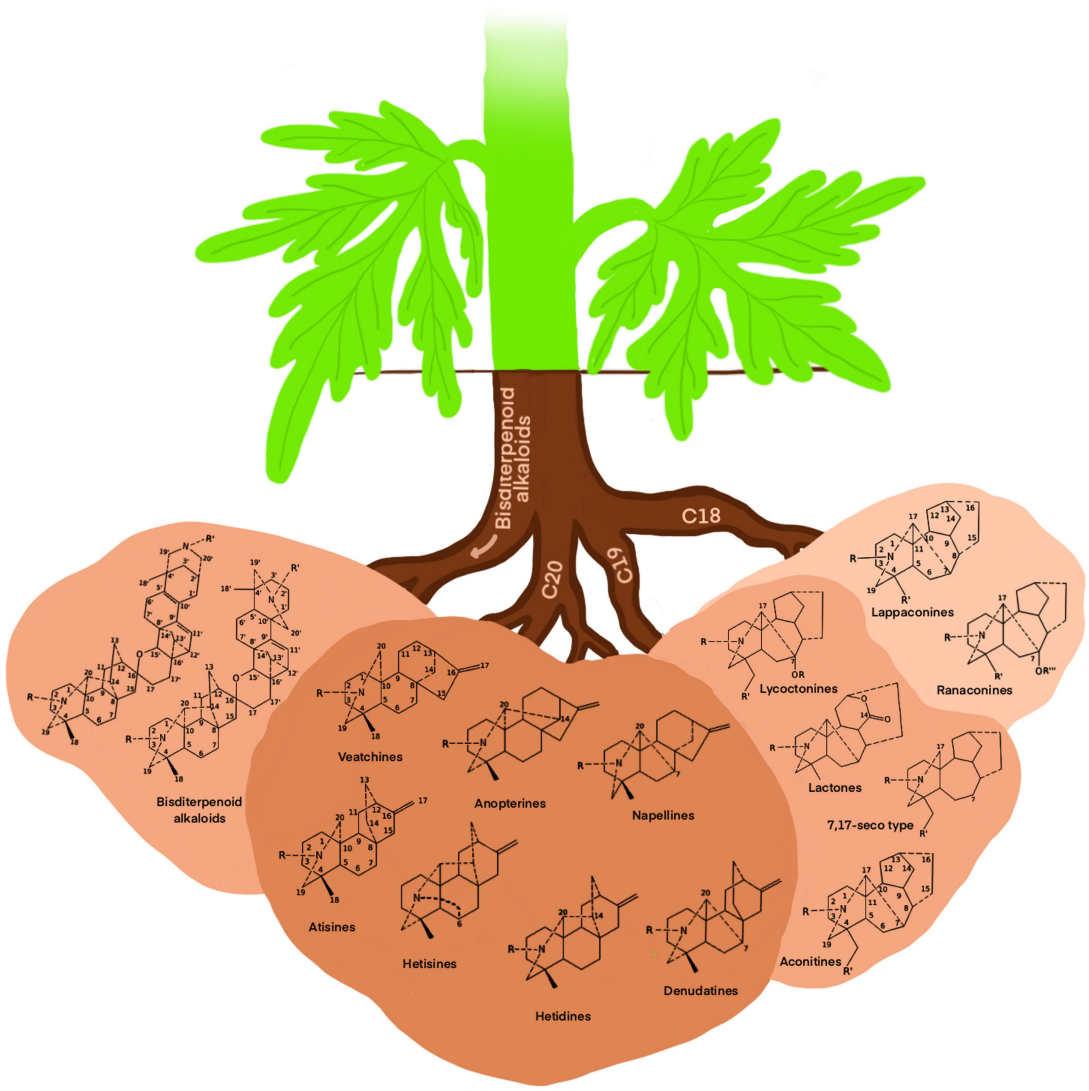
Cartoon diagram of skeletal types of diterpenoid alkaloids secreted by *Aconitum* spp. utilizing available information (Xiao et al., 2006).

Owing to their significant biological activity, toxicity, and effect on the nervous system and cardiac health, alkaloids are the secondary metabolites of interest in the case of *Aconitum*. The major *Aconitum* toxic metabolites are aconitine, mesaconitine, and jesaconitine, which fall under steroid alkaloids. Due to increasing interest in *Aconitum*, other compounds such as flavonoids and carbohydrates have also been identified and studied in *Aconitum* plants (Hao et al., 2015b; Yin et al., 2019). *Aconitum* poisoning can occur due to improper administration of the plant extract because of their toxic diester diterpene alkaloids. Nowadays, high-tech chromatography techniques can monitor the presence of traces of toxic *Aconitum* metabolites, which are used as raw material to produce drugs or other medicinal concoctions. Several databases, such as KNApSAcK and LOTUS-DB, have an extensive list of *Aconitum* secondary metabolites. Broadly, *Aconitum* diterpenoid alkaloids fall into four skeletal types C18, C19, C20, and bisditerpenoid alkaloids (Xiao et al., 2006), further subdivided into 14 sub-groups as depicted in **Figure 5**.

## Biological properties and pharmacodynamics of *Aconitum*

*Aconitum* has been part of the traditional Chinese medicine system (TCM) for centuries. Its earliest record of being used almost two thousand years ago has been mentioned in the Chinese material Shennong Bencao Jing (Yin et al., 2019). It has been used for wind-dispelling and damp-drying, blood-activating, stasis-removing, interior-warming and cold-dissipating, traumatic injury, arthritis, neuropathic pain, stroke, and paralysis, cold and pain of stomach, gastroenteritis, menstrual disorder, ulcer disease sores, etc. (Hao et al., 2015c). *Aconitum*-based medicines have now made their way into the herbal medicine industry. For example, Fu Zi – also known as *Aconiti Lateralis Radix Praeparata*, is a TCM formula made from the lateral roots of *A. carmichaelii Debx* (Liu et al., 2020), used to relieve joint pain and treat rheumatic diseases (Köberl et al., 2013). The primary root is not used in preparations as it is highly toxic. Processed Fu Zi contains Yanfuzi (YFZ), Heishunpian (HSP), and Baifupian (BFP), which differ in their processing and are greatly desired to ensure low toxicity and clinical safety in decoctions (Sun et al., 2012). Another well-known herbal medicine, Shen-fu, is an injectable powder made of Fu Zi and Panax ginseng, used to treat heart failure and cerebral infarction (Zhang and Li, 2013). Rhizoma Zingiberis is used with Fuzi to reduce deadliness and improve the efficacy of curing heart failure (Peng et al., 2013). Combining *Herba Ephedrae* (Mahuang in Chinese) and Radix Aconiti Lateralis (Fu Zi) exerts an analgesic effect compared to individual alkaloids. When administered to rats, it was observed that the alkaloids had a high clearance rate from the body except for methylephedrine (Song et al., 2015). The ephedra and *Aconitum* alkaloids are excreted in urine and feces, respectively. Another herbal medicine, Chuan Wu is made of Radix Aconite (RA) and R. Paeoniae Alba (RPA). It is more toxic than Fu Zi due to RA, as it inhibits cytochromes. The presence of RPA in the cocktail nullifies the toxicity of RA as it increases the presence of monoester-diterpene alkaloids (MDAs) thereby increasing its efficacy. It mainly exerts its effect via voltage-gated Na^+^ channels. It produces a heightened membrane excitation in the cardiac and neural tissues producing a prolonged depolarized state during which the next cycle of repolarization is delayed, which ultimately leads to arrhythmia, palpitations, numbness, and paralysis in the upper and lower limbs of the body.

Alkaloids isolated from *A. laeve* are found to be acetylcholine esterase inhibitors (Ahmad et al., 2018) which may provide a lead for several new arenas in Alzheimer’s disease treatment. Alzheimer’s disease is characterized by the loss of acetylcholine-producing neurons and an increase in the breakdown of the remaining acetylcholine by the enzyme acetylcholine esterase (AChE). Hence, the disease progression can be delayed by reducing levels of AChE to allow the minimal acetylcholine present to work. Lycoctonine-type norditerpenoid alkaloid, swatinine-C 1 from *A. laeve*, is shown to reduce the activity of AchE (Ahmad et al., 2018). Three new compounds - 6β-Methoxy-9β-dihydroxylheteratisine; 1α,11,13β-trihydroxylhetisine; 6,15β-dihydroxylhetisine – isolated from *A. heterophyllum* were proved to have anti-choline esterase activity in a recent study (Ahmad et al., 2017). Higenamine isolated from *Aconitum* tubers is known to exert its effect on the cardiovascular system. Mesaconine ameliorates the left ventricular diastolic function and brings an inotropic effect as a part of mediating its protective effects. Anti-arrhythmic Acehytisine isolated from the root of *A. coreanum* aids in blocking effects on pacemaker currents, as observed in *Xenopus laevis* oocytes. The frequency of arrhythmia remarkably decreases due to repeated administration of aconitine over a long period (Hao et al., 2015a)*. A. baikalense* and *A. septentrionale* extracts are found to reduce inflammation, and their effects are comparable to those of nonsteroidal anti-inflammatory drugs currently on the market.

Pharmacodynamics is about how the body responds to a drug. The key focus of scientific research on *Aconitum* has been to understand the effect of diterpene alkaloids on the heart and central nervous system (Nyirimigabo et al., 2015), which we have discussed here:

### Cardiac effects

**Figure 6:**
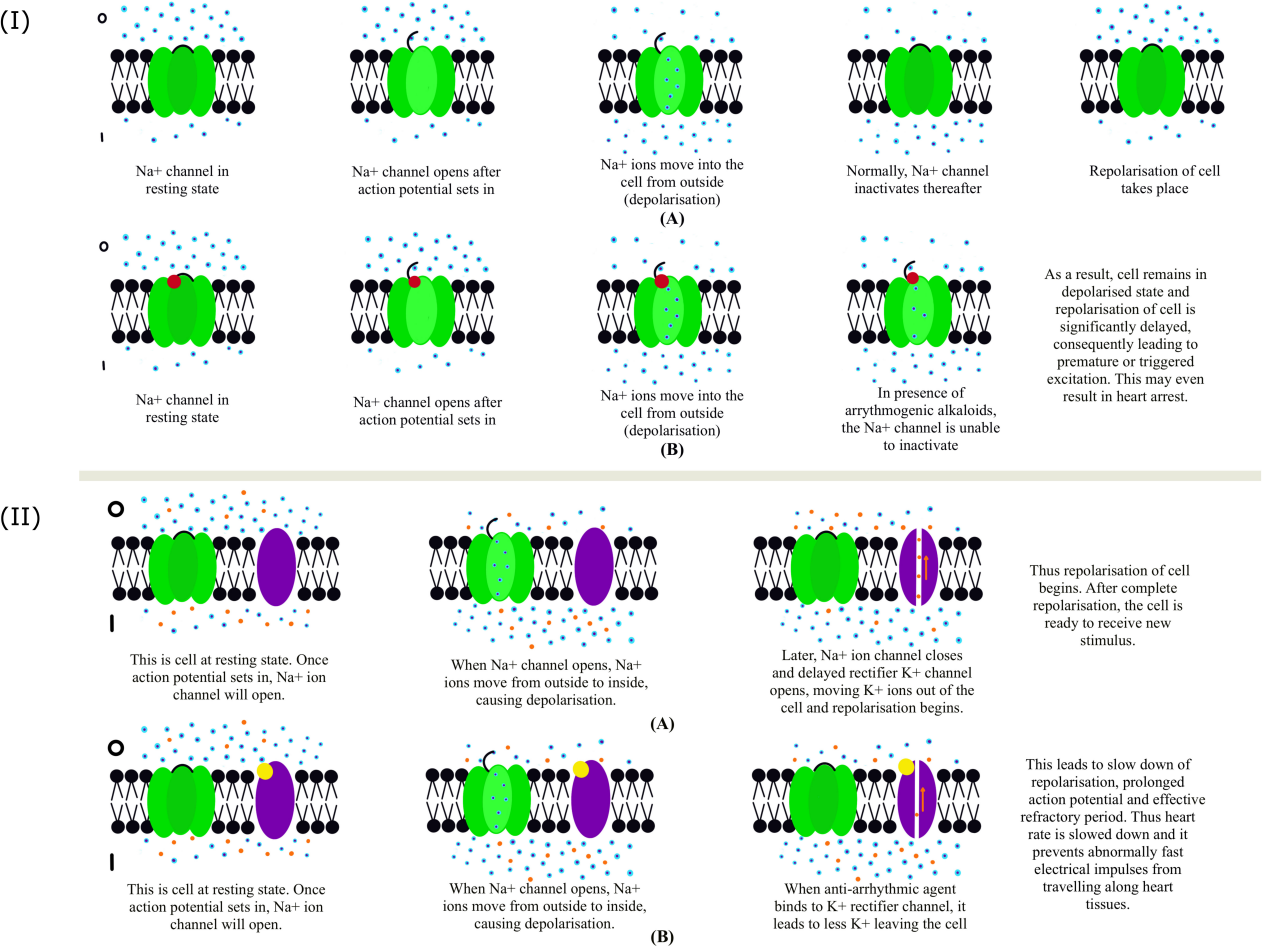
**(I)** Functioning of Na^+^ ion channel: **(A)** This panel represents normal functioning of Na^+^ ion channel in the absence of any arrhythmogenic agent. **(B)** This panel represents the abnormal functioning of the Na^+^ channel in the presence of an arrhythmogenic agent. Blue and red dots represent Na^+^ ions and arrhythmogenic agents, respectively. The resting membrane potential of a healthy myocardial cell is around -90mV. The inside of the cell is more negative in comparison to the outside. Also, the number of Na^+^ ions is higher on the outside, thus maintaining the polarized state of the cell. When there is leakage of Ca^+^ ions from the neighboring cells, the cell’s resting potential is disturbed. At one point, the threshold or action potential is reached, after which Na^+^ ion channels open and Na^+^ ions rush inside the cell from outside, thus increasing the membrane potential or depolarizing the cell. When the Na^+^ ion channel is inhibited, the cell returns to its normal polarized state. When an arrhythmogenic agent is present (panel B), it binds to the Na^+^ ion channel and prevents its inhibition, significantly delaying the cell’s repolarization phase. This may finally lead to in-excitability of the cell and, consequently, heart arrest. **(II)** Functioning of Na^+^ and delayed rectifier K^+^ channel: **(A)** The panel represents the normal functioning of Na^+^ and delayed rectifier K^+^ channel. **(B)** The panel represents the abnormal functioning of Na^+^ and delayed rectifier K^+^ channel in the presence of an anti-arrhythmogenic agent. Blue, orange, and yellow dots represent Na^+^ ions, K^+^ ions, and anti-arrhythmogenic agents. Na^+^ and K^+^ ion channels are shown in green and purple. After an action potential, inhibition of Na^+^ ion channel occurs, simultaneously opening the delayed rectifier K^+^ ion channel (IKr). Opening of IKr channel leads to the movement of K^+^ ions from inside of the cell to outside, thus making inner side of the cell more negative, or repolarization of the cell begins. In some cases, the impulses for heart contraction find alternate pathways other than the normal Sinoatrial (SA) node pathway. Consequently, the heart beats fast and irregularly. To deal with this, anti-arrhythmogenic agents are used, which slow down the heart rate by binding to IKr and reducing the number of K^+^ ions that move out of the cell, thus delaying repolarization (panel B). It does not completely inhibit the channel function. Thus, the rise of abnormally fast electrical impulses along the heart tissues (arrhythmia) is prevented.

The effects of diterpene alkaloids on the heart and the central nervous system are mainly because of their ability to interact and influence the activity of voltage-gated Na^+^ channels (Dzhakhangirov et al., 2005). Regarding their cardiac effects, the action of *Aconitum* alkaloids can be classified into arrhythmogenic and anti-arrhythmic alkaloids (**Figure 6**).

#### Arrhythmogenic alkaloids

When a cardiac cell is at resting potential, and an action potential is triggered in a neighboring cell, voltage-gated sodium channels open, and sodium ions rush inside the cell from outside (along the chemical gradient), causing rapid depolarization. In normal cells, these voltage-gated sodium channels become inactivated after rapid depolarization (**Figure 6A**). However, arrhythmogenic alkaloids interact with the voltage-gated Na^+^ channels in such a way that they inhibit their inactivation after this step (Ameri, n.d). Due to this, the cell remains depolarized, delaying the final repolarization phase of action potential in the heart cells. Because of premature or triggered excitations, the in-excitability of cells may even lead to heart arrest (Ameri, n.d). One of the most active, highly toxic, and fatal arrhythmogenic alkaloids, Aconitine (AC), has a high affinity for the open state of the Na^+^ channel at neurotoxin binding site 2. AC and related compounds are the main alkaloids of several *Aconitum* spp. used in traditional medicine preparations. The arrhythmogenic alkaloids have an AC skeleton, and their cardiotoxicity is attributed mainly to its substituents, namely β-OH on C-13, α-aroyl on C-14, β-acetate on C-8, and a positively charged nitrogen atom. To neutralize its toxicity, the integrity of these three substituents needs to be dismantled, which interestingly reverses the quality of the AC skeleton alkaloids.

#### Anti-arrhythmogenic alkaloids

Unlike arrhythmogenic compounds, anti-arrhythmic alkaloids are based on various diterpene skeletons. It also functions contrary to arrhythmogenic alkaloids as the anti-arrhythmogenic alkaloids inhibit the voltage-gated sodium channel and block the delayed rectifier potassium ion channel whose function is to leak potassium ions out of the cell and thus make the cell membrane potential more negative (**Figure 6B**). The arrhythmogenic and anti-arrhythmogenic alkaloids are competitive antagonists of the Na^+^ channel. The most active anti-arrhythmogenic diterpene alkaloids are the C18 diterpene alkaloids, and their standard structural features are - the presence of methoxy groups on C-1, C-14, and C-16; acetylanthranilic or anthranilic acid on C-4; and an OH on C-8 (Dzhakhangirov et al., 1997). Lappaconitine, a C18 diterpene alkaloid, irreversibly blocks open human heart Na^+^ channels, thus having anti-arrhythmogenic properties (Wright, 2001). Studies on its effect on isolated guinea pig hearts showed a decreased spontaneous beating frequency (Heubachl and Schüle, n.d). Other alkaloids with high anti-arrhythmogenic activity, such as 14-benzoyltalatisamine have a benzoyl group on C-14. 14-benzoyltalatisamine is a potent and selective blocker of the delayed rectifier K^+^ channels and thus plays a vital role as an anti-arrhythmic agent, given the crucial role of K^+^ channels in heart rate regulation. The most potent anti-arrhythmic action was observed with an aromatic residue on C-6, among the heteratisine type alkaloids such as 6-Benzoylheteratisine. From the napelline-type group, napelline, songorine, and their 1-benzoyl derivatives are the most active. The benzoyl derivative is essential for the alkaloids to influence the Na^+^ channel. Another anti-arrhythmic member belongs to Hetisine-type of the guanfu base series. In rats, Guanfu base A was shown to reduce the incidence of ventricular fibrillation induced by CaCl_2_ (Dong and Chen, 1995). LD50/ED50 ratio has been studied for several *Aconitum* alkaloids and compared with other anti-arrhythmic drugs (Dzhakhangirov et al., 2005). Some alkaloids, i.e., lappaconitine, N-Deacetyllappaconitine and 6-Benzoylheteratisine performed similarly or even better than previously existing drugs. The drug lappaconitine hydrobromide (allapinin) (Mazur et al., 1986) proved effective in clinical trials as a class I C anti-arrhythmic drug. Class I anti-arrhythmic drugs are the ones that bind to and inhibit the Na^+^ channel as their mode of action. Allapinin primarily prevents paroxysmal atrial fibrillations (Sokolov and Dzhakhangirov, 2002). Other promising alkaloids showing positive results in animal models, such as ‘Guanfu’ base A and 6-benzoylheteratisine are currently under clinical trials (Nyirimigabo et al., 2015). Guanfu base A is a promising anti-arrhythmic drug without severe side effects where it can cause QT prolongation.

### Other effects on cardiac health

Another *Aconitum* alkaloid Higenamine has cardiotonic properties, which have been studied on isolated rat atria revealing ionotropic and chronotropic results helping aorta relaxation. The ionotropic effect refers to the alteration in force or energy of heart contraction, whereas chronotropic refers to the heartbeat rate. It inhibits epinephrine, ADP, or collagen-induced platelet aggregation in platelet-rich plasma. Due to this, the blood pressure in rats lowers, whereas the recovery rate is increased in the acute thrombosis model (Wong et al., 1997). The implication of raw and processed aconite root extract on isolated toad cordis improved its contractile force (Wang W. et al., 2009). Metabolites mesaconitine, hypaconitine, and beiwutinine from *A. carmichaelii* were tested for their effect on isolated perfused bullfrog hearts. Mesaconitine improved ionotropic effect and left ventricular diastolic function (Jian et al., 2012). In another study, Acehytisine from the roots of *A. coreanum* was studied for its cardiovascular effects on oocytes of *Xenopus laevis*, revealing that acehytisine had a blocking effect on pacemaker currents in sinoatrial (SA) node cells and human HCN4 channels expressed in the oocytes (Fan et al., 2012). HCN4 channel is a sodium/potassium hyperpolarization-activated cyclic nucleotide-gated channel and functions to allow potassium and sodium ions to flow into cells of the SA node. This ion flow is often called the pacemaker current. Guanfu base A and Guanfu base G from *A. coreanum* block hERG channel current (Huang et al., 2012). hERG gene is a human ether-a-go-go-related gene that codes for a pore-forming K+ delayed rectifier channel subunit. The alkaloid aconitine from *A. napellus* subsp. *firmum* blocks the GIRK channel but shows no effect on hERG channel (Kiss et al., 2013). The function of GIRK channel is to hyperpolarize neurons as a response to many G-protein coupled receptors activation. Thus, these channels control the excitability of neurons through GIRK-mediated self-inhibition and slow synaptic potentials. The activity of aconitine thus hints towards its nature to induce cardiotoxicity. Radix Aconiti lateralis preparate (Zhi Fu Zi) exerted hypotensive effects in portal vein ligation rats (Lin et al., 2007). The aconite tuber constituents also increase plasma nitrite and nitrate levels, leading to NO levels and overall affecting peripheral vascular function (Yamada et al., 2005).

### Effect on nervous system

Similar to the effects of *Aconitum* alkaloids on the cardiac cells, their clinical impact on the nervous system is also attributed to Na^+^ channels and their activation. Those alkaloids have antinociceptive properties suggesting that they can activate the voltage-gated Na^+^ channel, depolarizing the neurons permanently and, therefore, blocking neuronal conduction (Friese et al., 1997; Polyakov et al., 2005). Recent studies have indicated their analgesic effect was reduced in opioid µ-receptor knockout mice (Murayama et al., 1984). Some *Aconitum* alkaloids lead to the inhibition of noradrenaline uptake, which is proposed to have a similar mechanism to the analgesic activity of alkaloids (Seitz and Ameri, 1998). Although using traditional *Aconitum* drugs as an analgesic is well established, it poses a severe risk because of the associated toxicity. Even though the *Aconitum* tubers are processed before their use as raw material in commercial production, the analgesic effect of *Aconitum* decreases to some extent, but still, it is not entirely safe from the poisoning risk.

### Anti-epileptiform effect

Anti-epileptiform activity of *Aconitum* alkaloids is attributed to their property to inhibit the Na^+^ channels since the sodium channels are known to be involved in the pathophysiology of epilepsy, wherein the nerve cell activity is disturbed, causing seizures. Na^+^ channel-blocking compounds such as lappaconitine have been shown to inhibit the experimentally induced epileptiform activity while sparing regular neuronal activity (Ameri, n.d). An aromatic substituent is essential for anti-epileptic activity; examples of *Aconitum* alkaloids that possess an aromatic substituent and inhibit rat hippocampal excitability are 6-benzoylheteratisine, 1-Benzoylnapelline, lappaconitine, and 14-Benzoyltalatisamine. These compounds are more potent than heteratisine, napelline, lappaconitine, and talatisamine (Ameri, 1997a; Ameri, 1997b; Ameri, 1997c). Regarding anti-epileptiform activity of *Aconitum* alkaloids, some compounds such as AC and 3-Acetylaconitine suppress epileptiform activity of nerves and ultimately stop the regular neuronal activity (Ameri et al., 1996; Ameri, 1997b). The effect of complete suppression of neuronal activity can be combated to some extent with the help of 6-Benzoylheteratisine, which inhibits the Na^+^ channel (Ameri and Simmet, 1999).

### Effect on nicotinic acetylcholine receptors

A well-known neurotoxin, i.e., A-bungarotoxin binds to the outer region of nAChRs (Nicotinic Acetylcholine Receptor). A diterpene alkaloid named Methyllycaconitine, first isolated from *Delphinium brownii* was found to competitively inhibit the binding of α-Bungarotoxin to the rat brain membrane and decrease the channel opening (Ward et al., 1990). It is also one of the most selective antagonists of brain α7-type nAChRs (Alkondon et al., 1992). Contrary to α-Bungarotoxin, methyllycaconitine is a small molecule capable of crossing the blood-brain barrier, and thus, alkaloids that interact with α7-type nAChRs have an essential role in neurobiological research (Navarro et al., 2000). This compound was also shown to have protective effects against β-amyloid induced neurotoxicity *in vitro* and requires more research in treating Alzheimer’s disease. NAChRs are vital targets in the drug discovery field because of their role in several disorders such as Tourette’s syndrome, anxiety, depression, smoking cessation, and irritable bowel syndrome (Leonard et al., 2000; Navarro et al., 2000; Tucci et al., 2003; Senn et al., 2022). Ligands of this receptor that specifically bind to nAChR are particularly significant for drug discovery. Such ligand molecules can also serve as imaging agents in diagnosing or prognosis of diseases such as Alzheimer’s disease, Parkinson’s disease, and Schizophrenia, given the observation that patients with these diseases possess significantly reduced numbers of nAChRs (Otvos et al., 2019).

### Anticancer chemodiversity of *Aconitum* spp.

Various metabolites from *Aconitum* spp. have been studied on cell lines and animal models for their anticancer properties (Hao et al., 2017). Effect of *A. carmichaelii* metabolites such as Aconitine, mesaconitine, hypaconitine, and oxonitine have been studied on the human liver cancer cell line HepG2 revealing potent growth inhibition of HepG2 cells. This property is attributed to the presence of ester groups on these diterpenoid alkaloids (Gao et al., 2012). BC1, an aconitine-containing agent, inhibited the growth of a solid form of Ehrlich’s carcinoma in mice by 77.3% (Garmanchouk et al., 2005). Atisine-type alkaloids on A549 human lung adenocarcinoma cell line inhibit the proliferation of these cells putatively via inhibition of matrix metalloproteinase – 2 and 9 (Xu X.-F. et al., 2010). The parent alkaloid having hydroxy groups at C-11 and C-15 positions has a lesser impact than the alkaloid having C-11 and C-15 positions replaced by the acyl group (Wada et al., 2011). The proposed action mechanism inhibits the cell line through G1 arrest in the latter case. Amide alkaloids from *A. taipeicum* exhibited cell growth inhibition activity on HL-60 leukemia cells and anti-tumor activity on K562 myelogenous leukemia cell line (Xu Y. et al., 2010). Taipeinine A, C19 diterpenoid alkaloids showed anticancer activity against HepG2 human liver cancer cell lines by blocking the cell cycle at the G1/S phase. The high dosage-induced apoptosis of tumor cells was proposed to be because of the upregulation of protein expression of Bax and caspase 3 along with downregulation Bcl-2 and CCND1 (Zhang et al., 2014). Bax is a pro-apoptotic protein, while caspase-3 is vital in the execution phase of apoptosis. CCND1 gene encodes the cyclin D1 protein, which promotes cell progression from G1 to S phase of cell cycle. Several non-diterpenoid alkaloids from *Aconitum* such as Neoline, 8-O methylcolumbianine, lycoctonine, browniine, delphatine, deacetylajadine, etc., were found to possess selective toxicity against cancerous cells via ATP production inhibition (De Inés et al., 2006). Alkaloids from *Aconitum* have also shown anticancer activity against A172 glioblastoma cell line (Wada et al., 2007).

### Toxicosis instigated by *Aconitum* alkaloids

*Aconitum* has cardiotoxic and neurotoxic effects because of diverse toxic alkaloids such as aconitine, mesaconitine, and hypaconitine; however, still, *Aconitum* species are consumed as a food material in some parts of the world. Consumption of aconitine, even in a minute amount or the raw plant, can lead to toxicosis. Toxicosis occurs mainly because of either misidentification, overconsumption, or improper processing of the plant material before consumption. From 2013 to 2018, there were over 5000 reported cases of *Aconitum* poisoning in China, with a fatality rate of 2.4%. Some instances of Aconitum poisoning are as follows:-

- *Aconitum ferox* plant poison, which contains the highly deadly alkaloid pseudaconitine, was used to kill Lakhvinder Cheema on January 27, 2009, in West London, by his former lover, Lakhvir Kaur Singh. Singh earned the moniker “The Curry Killer” due to the dish to which the poison was put (BBC, 2010).
- By accidentally ingesting a homemade infusion, which was later determined to be an *Aconitum* preparation that the patient had been using as a topical painkiller, a 54-year-old Chinese male was reported to have decreased level of consciousness, hypotension and had a cardiac arrest with pulseless ventricular tachycardia (Bonanno et al., 2020).
- Customers got sick at the Toronto-based Delight Restaurant & Barbeque after eating a dish. Hospitals in the area received patients with symptoms mimicking aconite poisoning. The accidental poisoning, according to officials, was brought on by a spice product contaminated with aconite (BBC, 2022).

Thus, it is crucial to rapidly diagnose the poisoning event and identify the food material to avoid the improper use of *Aconitum* materials. As there is no specific antidote, the primary treatment for poisoning is only via supportive care. Activated charcoal gastric lavage may be helpful. Techniques like hemodialysis or extracorporeal filtration are less effective for treating cardiotoxic plant poisoning (Magnani and Woolf, 2017; Jha and Padmaprakash, 2018). Antiemetic medications make it simple to treat nausea and vomiting. Rapid detection of seizures, cardiac arrhythmias, and hypotension can save lives. In a case study by Jesrani et al., the patient saw improvement with amiodarone and returned to normal sinus rhythm, highlighting the medication’s effectiveness in treating arrhythmias brought on by aconitine toxicity (Jesrani et al., 2022). For easy identification, researchers have recently identified a 23-bp genus-specific nucleotide sequence that can detect the presence of *Aconitum* traces even from a sample of highly degraded DNA, such as that found in processed food samples (Wang et al., 2022).

### Metabolism of *Aconitum* alkaloids

Despite containing diverse poisonous compounds, several *Aconitum* spp. has been used in TCM for over 2,000 years, among which *A. carmichaelii Debx* is the most used (Zhao et al., 2021; Yang et al., 2022). Comprehensive information about the therapeutic index of its alkaloids is unavailable, which has led to several cases of poisoning and death. Therefore, it is essential to study its route of absorption in the body after administration, as several of its herbs are consumed orally. Diester-diterpene alkaloids (DDAs) such as aconitine (AC), hypaconitine (HA), and mesaconitine (M), as well as monoester-diterpene alkaloids (MDAs), such as benzoylaconine (BAC), benzoylhypaconine (BHA), and benzoylmesaconine (BMA), make up the active *Aconitum* alkaloids in Fu Zi. Diverse factors, including the drug’s complex components and prescribed amounts, must be examined to ensure that Fu Zi is used therapeutically and safely. How to efficiently use medicinal plants or their secreted secondary metabolites has been a constant point of discussion. Sometimes, these alkaloids should be combined with multiple extracts instead of refined products. However, for this, one major limiting factor is to study their interaction with other herbs which are a part of the prescribed concoction. Another point of view is to consume purified alkaloids directly as the presence of other compounds in the extract might enable the transport and absorption of alkaloids can be significantly influenced by co-occurring components; for example, a crude extract of the Rooibos plant was proven to facilitate the passage of paeoniflorin flavonoid through the gut epithelial cells (Liu et al., 2012). Aconiti Radix Cocta gel, Aconiti Radix Cocta, and Paeoniae Radix Alba gel have been widely used in TCM. Combining these two herbs shows increased efficacy and reduced toxicity (Li et al., 2016). Lipid carriers containing the alkaloids of *A. sinomontanum* injected transdermally via a microneedle have shown to have several benefits such as reduced paw swelling, inflammation, and pain, better regulated immune function in adjuvant arthritis, and improved arrhythmia as observed in rats (Guo et al., 2017). The use of lipid carriers for sustained release of the alkaloids mediates a more significant therapeutic effect. A nasal spray that can deliver the alkaloids through the mucosa is yet to be developed.

### Metabolism in the gut

Intestinal absorption entails moving the substances from the apical side to the basolateral side of the intestinal cells. Caco-2, the human colon epithelial cancer cell line, is the most used cell line to study intestinal absorption of drugs and other compounds. In the gut, Aconitine (AC) outflux is more than influx due to p-glycoproteins on the apical membranes of the intestinal epithelial cells. The multi-drug resistance gene (Mdr1a) encodes for p-glycoprotein, an efflux pump of the ABC transporter family of drug transporters involved in AC efflux (Ho et al., 2003). The absence of p-glycoproteins causes a large amount of AC influx, eventually causing death. AC administration induces P-glycoproteins, which is correlated with toxicity. AC can induce CYP3A4/CYP206 isoforms which are involved in AC metabolism. On heating, AC breaks down to form benzoyl aconine (BAC) and aconine via hydrolysis, further reducing its toxicity. P-glycoproteins reduce the absorption of AC and reduce its poisonousness in animals in the order of aconitine (less absorbed), benzylaconine, and aconine (highly absorbed). AC gets absorbed percutaneously and enters the blood, leading to intoxication. The liver and kidney tissues have more *Aconitum* alkaloids distributed in them than in the heart and cerebrum (Ito et al., 2000). The order of aconite levels in body fluids is highest in urine, followed by bile, gastric contents, heart, and blood.

#### Impact of *Aconitum* alkaloids on gut microbiota

Intestinal biotransformation of Aconitine involves the breakdown of Aconitine by gut microorganisms into twenty different metabolites such as mono-ester *Aconitum* alkaloids, diester aconitines, and lipo-alkaloids by deacetylation, dehydroxylation, demethylation and esterification steps (Zhao et al., 2008). However, the enzymes catalyzing these steps have not been reported yet. Intestinal microorganisms have been found to convert AC to lipoaconitines, 8-O-Oleoylbenzoylaconine and 8-O-Palmitoylbenzoylaconine (Hao, 2018). 14-O-Acetylneoline isolated from the roots of *A. laciniatum* shows anti-colitis and anti-inflammatory activities as it reduces the production of proinflammatory cytokines (Wangchuk et al., 2015). *Aconitum* is mainly used in traditional Chinese medicine as it is cold alleviating (Makino et al., 2009), but the mechanism to promote thermogenesis remains unclear. Gut microorganisms play a significant role in thermogenesis (Xiao and Kang, 2020). Treating rats with Aconite aqueous extract increased the number of *Ruminococcaceae, Desulfovibrionaceae, and Enterococcaceae* bacterial families, which are known to be involved in browning adipose tissue, which further increases thermogenesis (Liu et al., 2021). Additionally, it promoted the enrichment of *Lactobacillus* and *Prevotella*, which increase the expression of UCP1 (uncoupling protein 1) in adipose tissues, thereby defining their role in promoting thermogenesis.

Fuzi-Lizhong pill (FLZP), made of *A. carmichaelii Debx. (Fuzi), Zingiber officinale Rosc. (Ganjiang), Glycyrrhiza uralensis Fisch. (Gancao), Codonopsis pilosula (Franch.) Nannf. (Dangshen)*, and *Atractylodes macrocephala Koidz*, alleviates diarrhea-predominant irritable bowel syndrome by inhibiting *Bacteroidetes, Blautia, Turicibacter*, and *Ruminococcus torques* group and increases *Lactobacillus*, crucial intestinal bacteria, to restore immunity (Zhen et al., 2021). TCM also prescribes consuming aqueous root extract of *A. heterophyllum* to cure diarrhea. This root extract is non-toxic when consumed in the 2g/kg range. It prevents the loss of Na^+^/K^+^ ions and restores the Na^+^/K^+^ ATPase activity in the intestine, thereby increasing the absorption of fluids in the body. The roots of *A. heterophyllum* contain alkaloids, carbohydrates, phenols and flavonoids, tannins, and saponins. The alkaloids possess anti-diarrheal activity; carbohydrates are wound healing and energy providing; phenols and flavonoids act as antioxidants and scavenge free radicals; tannins denature proteins to form protein tannates, making the intestinal mucosa more resistant to loss of fluids. The root extract is not only anti-diarrheal but also anti-microbial, as it was found to inhibit the growth of bacteria responsible for causing pathogenic diarrhea (Prasad et al., 2014).

### Metabolism in the Liver

Cytochrome P450s (CYPs), one of the body’s defense systems, is vital in transforming drugs. The *Aconitum* alkaloids serve as substrates, inhibitors, or inducers of CYPs. The liver is responsible for detoxifying substances in the body and has CYP3A as the dominant hepatic CYP in the liver microsomes. AC acts as a substrate for CYPs. CYP3A in human intestine microsomes metabolizes diester-diterpene alkaloids (DDAs) and monoester-diterpene alkaloids (MDAs). A study of recombinant cytochromes such as CYP3A5 and 2D6 on AC breakdown revealed that the transformation of AC occurs via hydroxylation and di-demethylation reactions (Tang et al., 2011). Breakdown of diester-diterpene alkaloids into monoester-diterpene (MDA) - benzoylaconine (BAC), benzoylhypaconine (BHA), and benzoylmesaconine (BMA) mitigates *Aconitum* poisonousness and therefore, exerts a positive therapeutic effect. Bulleyaconitine A (BLA) obtained from *Aconitum bulleyanum* is widely used in TCM as an anti-inflammatory drug. It is transformed in the liver microsomes by CYP3A5 and 2D6 via deacetylation, demethylation, hydroxylation, and dehydrogenation-deacetylation (Hao et al., 2015a).

## Pharmacokinetics of *Aconitum* secondary metabolites

In simplest terms, ‘pharmacokinetics’ refers to what the body does to the drug or, in other words, the movement of drugs through the body. Several attempts have been made to understand the pharmacokinetics of *Aconitum* compounds (Tang et al., 2012). Aconitine and aconine were primarily excreted in the urine, whereas benzylaconine was eliminated in the feces (Zhang et al., 2016). Also, their metabolic stability studies suggested that BAC and ACN were more difficult to metabolize than AC. The tissue distribution experiments showed that the alkaloids were distributed across all organs; however, the distribution rate of AC was slower than BAC and CAN (Zhang et al., 2016). When pure aconitine and fuzi (processed daughter roots of *Aconitum carmichaelii)* were administered orally and intravenously to rats in single and multiple doses (Tang et al., 2012), the bioavailability of aconitine remained low with no difference in their pharmacokinetic properties in single and multiple dosages. However, multiple doses of processed Fuzi extract led to an increase in the bioavailability of aconitine, thus resulting in higher chances of toxicity. Moreover, aconitine gets eliminated rapidly, indicating its low plasma protein binding ability (Tang et al., 2012).

## Herb-drug interaction

Traditional herbal products are being increasingly integrated into Western medicine because of their usage history and the notion that safety is ensured by “natural”; however, TCM still does not recommend using herbs with conventional drugs (Izzo, 2012). Consumers typically self-administer these drugs with traditional medications without consulting their doctor or healthcare professional. Due to the heterogeneity in herbal product composition, uncertainty around the causal components, and frequently limited understanding of their pharmacokinetics, evaluating the liability of herbal product interaction is difficult (Brantley et al., 2014). Such herb-drug combinations may have undesirable effects if the herbal product disturbs the function of transporters and/or enzymes that metabolize drugs. The main limitations of HDIs are as follows -

- Less importance is given to the effects of dose, regimen, and mode of medication.
- Information remains lacking for the main CYP and UDP-glucuronosyltransferase (UGT) enzymes in the less-studied medicinal plants.
- Requires more Information on P-glycoproteins (P-gp) and other drug transporters
- ABC transporter and solute carrier (SLC) superfamilies have many other transporters besides P-gp, organic cation transporters, and Glucose transporters, which await future investigations.

Herbs can alter the anticipated activity of prescription medicine, causing either undesired side effects or therapeutic failure, leading to significant pharmacokinetic and pharmacodynamic changes. Therefore, TCM prefers all concoctions to be made with either a single herb or a combination of two herbs and not with any drug (Wang et al., 2012).

## Herb-herb interaction

In TCM, HHIs are preferred over HDIs to achieve synergistic therapeutic effects. Several different combinations of herbs can be used with their unique benefit to regulate other targets. The main aim is to minimize the adverse effects of toxic ingredients and enhance the pharmacological potency of agents. Fu Zi is an excellent TCM formula that consists of two herbs isolated from lateral roots of *Aconitum carmichaelii Debx* and is widely used to relieve joint pain and treat rheumatic diseases (Sun et al., 2012). The primary plant root is not used in preparations as it is highly toxic. Processed Fu Zi contains Hei-Shun-Pian (HSP). Rhizoma Zingiberis is also used along with Fuzi to reduce deadliness and improve efficacy. When administered, H. Ephedrae - Radix Aconiti Lateralis (Fu Zi) shows a high clearance rate from the body except for Hypaconitine. Another formula, Shen-fu, made of Fu Zi and *Panax ginseng*, is an injectable powder potent in treating heart failure and cerebral infarction. The ephedra and *Aconitum* alkaloids are excreted in urine and feces, respectively. Chuan Wu made of Radix Aconite (RA) and Radix Paeoniae Alba (RPA), is more toxic than Fu Zi due to the presence of RA as it is involved in the inhibition of cytochromes (Bi et al., 2014). The occurrence of RPA in the cocktail nullifies the toxicity of RA, thereby increasing overall efficacy. Compared with single-herb extracts, using medicine herb pairs leads to a prolonged residence time and delayed elimination of *Aconitum* alkaloids, increasing the risk of drug accumulation (Hao, 2018).

## Conservation strategies of *Aconitum* spp.

Understanding the genetic structure of a species at a population level is necessary to develop efficient conservation strategies (Chung et al., 2011). The endangered status of *Aconitum* spp. and its several health benefits warrant proper conservation of *Aconitum* spp. The geographical sites at which it is found should establish field stations to cultivate and store the plant’s germplasm. *Ex situ* conservation involves the establishment of nurseries, plantations, and medicinal plant gardens. It also involves setting up seed banks and gene banks. However, *ex-situ* conservation of *Aconitum* is failing due to poor seed availability, lack of isolation of superior germplasm, and the presence of polysaccharides and phenols in the plant (Srivastava et al., 2010). This method can be more impactful by the cold stratification technique, which, under moist conditions and plant growth hormones (auxin, gibberellic acid), nitrates, nitrites, and cyanide help break seed dormancy (Ali et al., 2019). Cyanide has successfully reduced apple seeds’ seed dormancy (Bogatek et al., 1991). Seeds of *A. heterophyllum* could be germinated upon treatment with a lower temperature and 0.5 mg/l of Auxin hormone (Pandey et al., 2000). *In-situ* conservation is a better strategy as it involves growing the plant in its native niche to enhance the existing population. It consists in exploring a given area of medicinal plants, identifying them, and marking the whole site as conserved. Plant tissue culture is another poignant strategy to preserve an endangered species, as a small part of the plant is sufficient to regenerate the whole plant. Mass regeneration of *Aconitum* via tissue culture or cultivating them on a larger scale can also benefit the herbal medicine industry. Micropropagation too has been applied to *Aconitum* cultivation, where multiple shoots and roots were generated using the tip tissue culture method. In an alternative strategy, *Agrobacterium rhizogenes*, a gram-negative bacterium is allowed to infect the *Aconitum* tissue producing hairy roots which can then be used to grow the plant further (Giri et al., 1997).

Conservation strategies for *Aconitum* should also involve a detailed study of its morphology, cytology, and ecology, as this information aids in designing a better conservation plan and increasing the understanding of the species. All morphological variability parameters, such as the plant structure, number of shoots and leaves, rhizome dimensions, plant height, leaf and flower dimensions, and number and size of seeds, should be considered altogether (Jeelani et al., 2015). The meiosis process in plants also requires cytological investigations since aberrant meiosis is a sign of ecological stress (Fuchs et al., 2018). Abnormal meiosis includes cytomixis, chromosomal stickiness, chromatin fragmentation, unoriented bivalents, formation of chromosomal bridges, and chromosomal laggards (Jeelani et al., 2015). Such meiotic abnormalities give rise to nuclei of different sizes, rendering them sterile, which reduces the viability of the pollen, thereby creating morphological and genetic variations as observed in populations of *A. heterophyllum* (Jeelani et al., 2015). Ecological studies have three parameters – 1) quadrat data aids in determining the frequency, abundance, and density of a species; 2) the abundance-to-frequency ratio aids in understanding the distribution of the species, which can be randomly and uniformly distributed or remain clustered; and 3) the importance value index highlights how dominant a species is in each area. *A. heterophyllum* plants grow in shady alpine areas, with 90% of the population randomly distributed. The plant population at higher altitudes is stunted in growth, with fewer leaves and more flowers and seeds (Jeelani et al., 2015). It has also been reported that climate change caused by global warming (experimented using elevated CO2 exposure) can reduce secondary metabolite production and antimicrobial potency against various pathogens in *A. balfourii* and *A. heterophyllum* overall, which may impact their trade, survival, and medicinal application (Chandra et al., 2022).

### *In vitro* propagation of *Aconitum* spp.

Several *Aconitum* spp. are on the IUCN list for endangered species. Given the medicinal potential and growing interest of the scientific community in *Aconitum*, developing strategies to conserve them has become crucial. Although rules and regulations regarding its collection are in place, it is not enough to revive them. Thus, several research groups have attempted to use *in vitro* propagation to find methods to grow them successfully in the laboratory and take them to their natural habitat. These efforts have been tried on various *Aconitum* spp., such as *A. violaceum, A. heterophyllum, A. carmichaelii, A. balfourii, A. ferox*, etc. A group of researchers from Sikkim has published their work on *in vitro* propagation of *A. ferox*, an *Aconitum* sp. sporadically present in the Sikkim-covering ranges of the Himalayas, where its unchecked use and overcollection have made it vulnerable. Moreover, its prolonged seed germination rate makes its conservation strategies more complicated. Researchers have established *in vitro* propagation method for *A. ferox* using root tip explants that show a 70% survival rate after successful acclimatization in *ex vitro* conditions (Singh et al., 2020). Similarly, Pandey et al. sprouted axillary buds *in vitro* and used the small leaf segments obtained from them to develop a technique for micropropagation of *A. balfourii* Stapf (Pandey et al., 2004). Shooting and rooting were also successfully achieved, followed by plantlets transplantation in a greenhouse chamber where plant growth was observed.

## Importance of chloroplast genomics in DNA barcoding

Many *Aconitum* spp. is renowned for their medicinal properties and are traded heavily unlawfully. A lack of pharma-forensic expertise and tools for accurately identifying species make this trade hazardous for consumers as they are unaware of which herbal or non-herbal material they are ingesting. No complete/draft nuclear genome has been reported for any *Aconitum* spp., making it a significant area of research to understand their diversity, physiology, evolution, and breeding. Molecular markers approach instead of the traditional ones such as organoleptic methods (identification by the senses: taste, sight, smell, and touch), macroscopic and microscopic methods (identification by shape, color, and texture), and chemical profiling (e.g., TLC, HPLC-MS, MALDI-TOF, HPLC-UV) can indeed be relied upon due to its higher discriminatory power (Techen et al., 2014). The technique for identifying biological specimens using short DNA sequences is called DNA barcoding. Initially, for the first time in 2003, the 5’ end of cytochrome c oxidase 1 (CO1) from the mitochondrial genome was considered as a potential DNA barcode for not only identification of species in animals but also to define species boundaries, flagging new species and species delimitation (Hebert et al., 2003). However, using the mitochondrial region as a potential DNA barcode in plants is unsuitable due to the slow evolution rate and meager substitution rates. Due to these shortcomings in the mitochondrial genome in plants, the search for plant barcodes was extended to chloroplast and nuclear genome, which shows high substitution rates. The Consortium of the Barcode of Life Plant Working Group (CBOL) evaluated seven different loci in the chloroplast genome across the plant kingdom. It proposed rbcL and matK combination as plant barcodes as the combination provides high discriminatory power (Hollingsworth et al., 2009). The rbcL gene provides high universality but less species resolution, whereas matK shows high species resolution but less universality (Cameron et al., 1999; Singh et al., 2012). Therefore, the Chinese Plant Barcode of Life (BOL) Group proposed the addition of nuclear ITS (Internal Transcribed Spacer) to the matK + rbcL combination as a plant barcode to achieve maximum identification rates for closely related species (Li et al., 2011). ITS region has also been widely used to identify toxic plants (Matsuyama and Nishi, 2011), including *Aconitum* spp. (Luo et al., 2005; Boroń et al., 2020). However, certain plants contain an inadequate variety of information to be identified using DNA barcodes. In those circumstances, the chloroplast genome should be utilized as a super-barcode to distinguish and identify plants and their closely related species (Wei et al., 2020). Because genetic variation is critical for plants, the ability to preserve their evolutionary potential, adapt to changing environments, and maintain genetic variation are primary goals of most endangered species conservation methods. At the species level, SSRs, also known as microsatellites, have a high polymorphism rate. As a result, they have also been widely used in population genetic and evolution studies as useful molecular markers (Wei et al., 2020).

**Figure 7:**
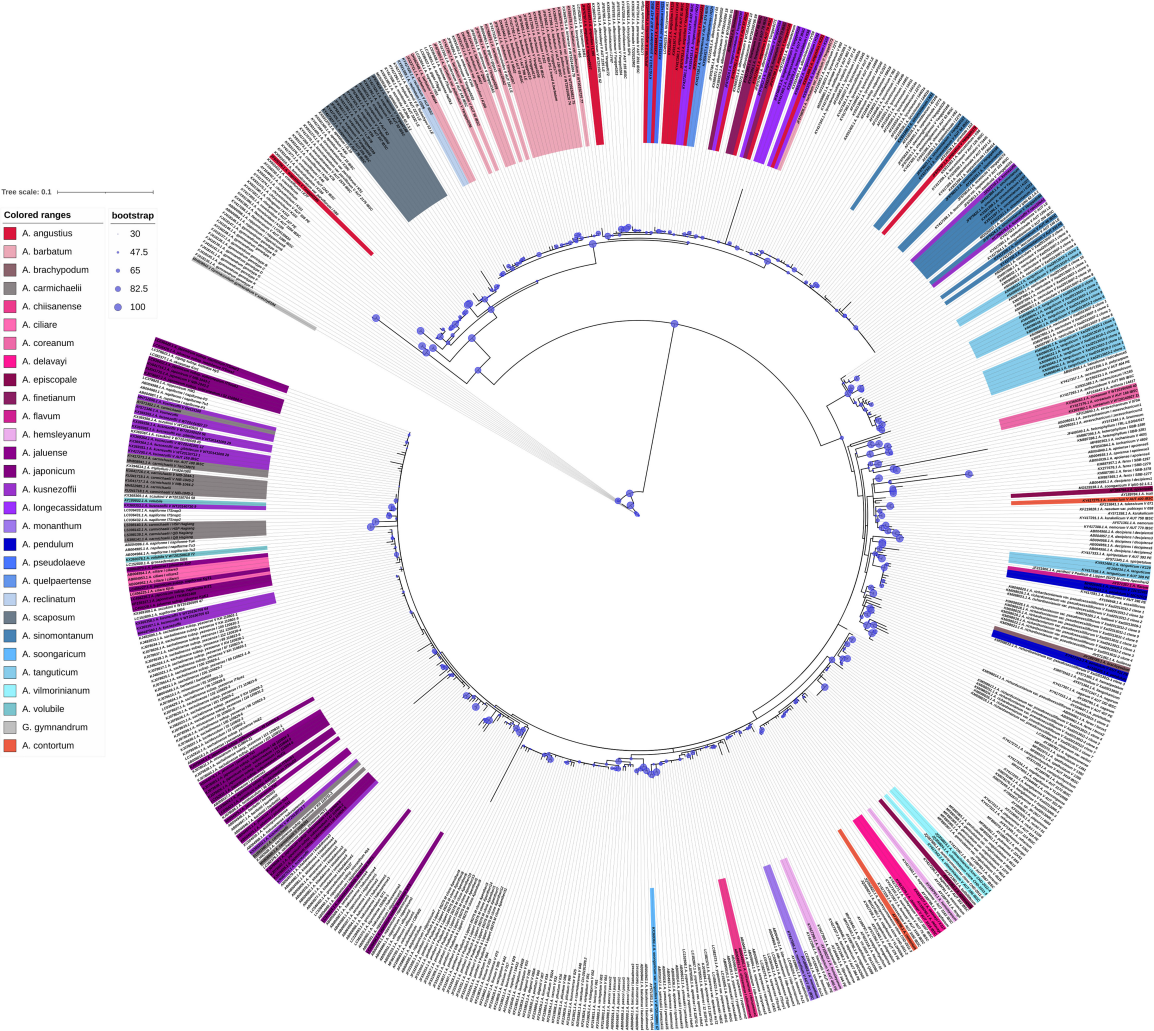
ITS Phylogeny: Maximum likelihood phylogeny depicting the *Aconitum* classification and taxonomic localization: ITS nucleotide sequences (>500 bp length) belonging to 631 *Aconitum* spp. and one outgroup *Gymnaconitum gymnandrum* were aligned using MUSCLE v3.8.1551. Sequence alignment was corrected and trimmed using trimal with “-gt 0.8 -st 0.001” parameters, which was further used to select the “Best Model selection” plugin in MEGA-X (Kumar et al., 2018). This master alignment was subjected as an input to build a maximum likelihood (ML) tree using RAxML v8.2.12 (Stamatakis, 2014) utilizing the best model and 100 bootstrap values. Tree visualization, taxonomy mapping, and bootstrap depiction (as shown in left side boxes) were done using iTOL v6 (Letunic and Bork, 2021).

**Figure 8:**
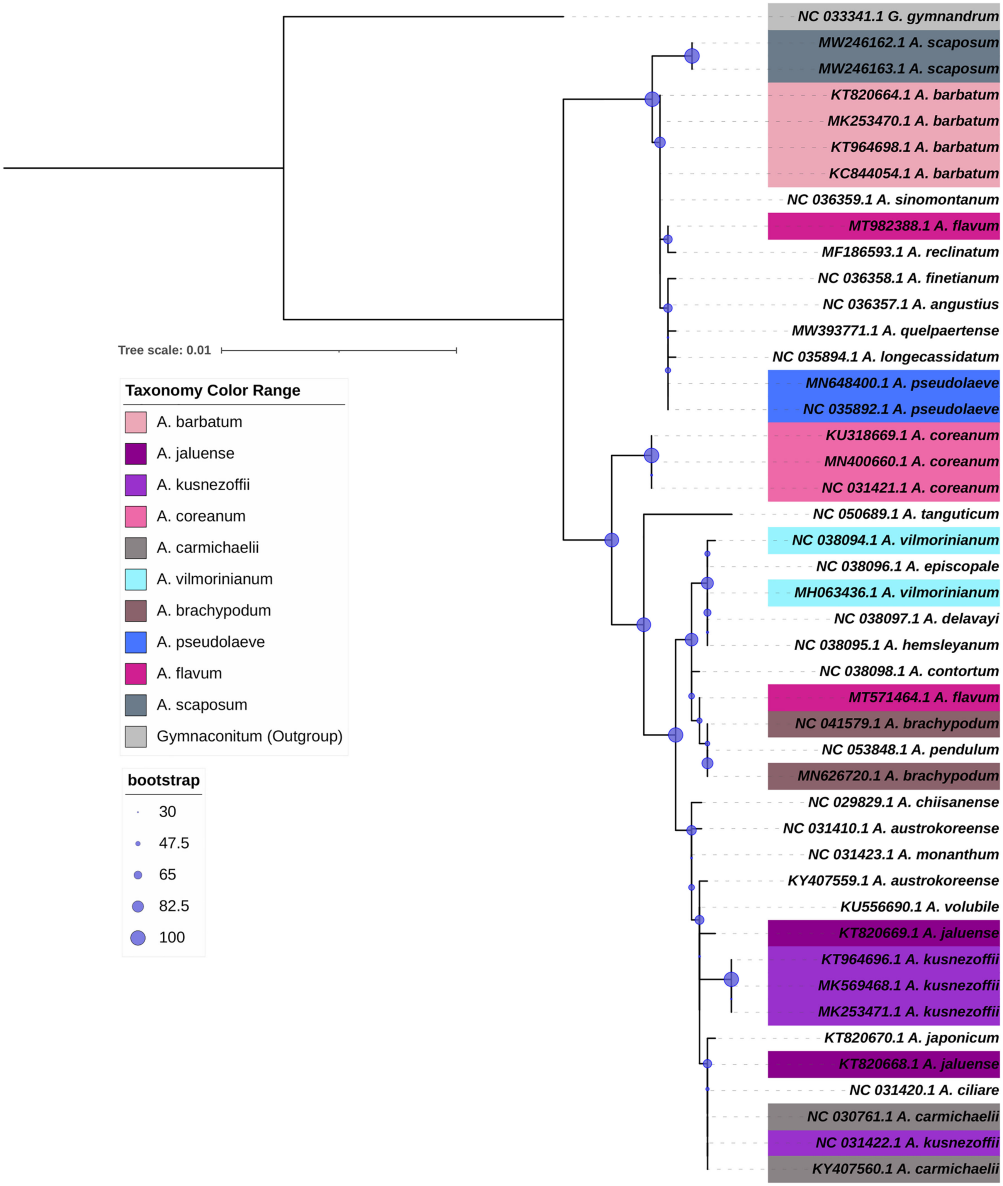
rbcL-matK Phylogeny: Two well-known marker genes, rbcL and matK, were extracted from 44 available complete chloroplast genomes of *Aconitum* spp. and one outgroup *Gymnaconitum gymnandrum*. Forty-five sequences from each protein sequence were aligned separately using MUSCLE v3.8.1551. Both alignment files were concatenated and further used to identify the “Best Model selection” plugin in MEGA-X (Kumar et al., 2018). This master alignment was subjected as an input to build a maximum likelihood (ML) tree using RAxML v8.2.12 (Stamatakis, 2014) utilizing the best model and 100 bootstrap values. Tree visualization, taxonomy mapping, and bootstrap depiction (as shown in left side boxes) were done using iTOL v6 (Letunic and Bork, 2021).

In the present study, we used both phylogenetic strategies, i.e., ITS (**Figure 7**) and concatenated housekeeping proteins present in the chloroplast (rbcL and matK) (**Figure 8**) for phylogenetic interference of *Aconitum* spp. This analysis revealed that both implemented phylogenomic strategies depicted similar relationships between different *Aconitum* spp., where most of the species were present in their respective clades, separated from other species. Broadly, concatenated housekeeping proteins-based phylogenies are considered more appropriate and trustworthy. As visible in housekeeping proteins-based phylogeny, there are two broad clades: the first one with *A. barbatum*, *A. flavum*, *A. scaposum*, *A. pseudolaeve*, etc., and the second one with *A. coreanum*, *A. vilmorinianum*, *A. brachypodum*, *A. kusnezoffii*, *A. carmichaelii*, etc. as prominent representatives for each clade. Here in housekeeping gene phylogeny, we could study only 44 chloroplast genomes belonging to 29 *Aconitum* spp. as chloroplast information of other spp. is unavailable in open-source data. As ITS information (632 sequences) was present for 164 unique *Aconitum* spp., we compared and cross-checked the taxonomic locations of representative species from the housekeeping phylogeny, confirming the robustness of ITS phylogeny. Based on this, we can extrapolate the putative relationship between diverse *Aconitum* spp. For example, *A. sachalinense* (26 ITS sequences) does not have any chloroplast data, but based on ITS phylogeny, it can be depicted that it is a close relative of *A. japonicum* and *A. jaluense*. *A. heterophyllum* has three ITS sequences and no chloroplast data. However, all three organisms are present in the same clade suggesting their closeness with each other. It can also be extrapolated that it is a close relative of *A. brunneum* (1 ITS sequence only) and *A. zeravschanicum* (3 ITS sequences only).

Another interesting example is *A. tanguticum* which has chloroplast data for one subspecies and 17 ITS representatives; chloroplast phylogeny suggests this organism to be a distinct member in itself as it is depicted as a separate branch in the second clade. ITS phylogeny also supports the same as 14 ITS sequences are present as a distinct clade away from the rest of clade 2; however, it is present here in close association with another member, *A. naviculare* (14 ITS sequences), suggesting both of them to be a close relative. Overall, we can argue that such housekeeping and ITS-focused phylogenies can help us understand the evolutionary relationship among the members under study, the *Aconitum* genus in the present case. Moreover, such analysis can also help us know which sequences have been wrongly classified and named; for example, three *A. tanguticum* ITS sequences are located separately from its prominent clade of 14 representatives suggesting their wrong nomenclature. We also identified such issues in our housekeeping phylogeny, i.e., *A. flavum* (MT982388.1) and *A. flavum* (MT571464.1) are present respectively in the first and second clade, which cannot be accurate and one of them must be wrongly named or identified. ITS phylogeny could also not distinguish it, as only one ITS sequence was in our data. With the availability of more chloroplast/nuclear/mitochondrial/ITS sequencing data in the future, we might be able to classify all *Aconitum* spp. more precisely.

## Transcriptome studies reveal upregulated genes in secondary metabolite pathways in roots

Secondary metabolite production in plants is tightly controlled regarding their expression and organelle localization. Several studies characterizing the metabolic extract of different *Aconitum* spp. have revealed their tissue-specific accumulation of various bioactive metabolites (Csupor et al., 2009). Therefore, transcriptomics analysis across various tissue types has been a unique methodology for studying the molecular components involved in the biosynthesis of these secondary metabolites and their functional classification and unraveling the molecular basis of various biological processes. These transcriptomics datasets can be used for genetic improvement of the *Aconitum* spp. either towards increasing the biomass or producing secondary metabolites. The first report of transcriptome analysis was reported for *A. heterophyllum* wall., (Pal et al., 2015) followed by transcriptome analysis reports on *A. Carmichaelii* (Rai et al., 2017; Zhao et al., 2018; Yang et al., 2020), *A. kusnezoffii* Reichb (Tang et al., 2021) and *A. vilmorinianum* (Li et al., 2021). These analyses resulted in the identification of molecular components involved in producing secondary metabolites in *Aconitum* spp. The plant part of significant interest within these studied organisms is their root due to the accumulation of diverse secondary metabolites such as aconitine and its derivatives mesaconitine, jesaconitine, and hypaconitine in the tuberous roots. Therefore, acknowledging the functional annotation of genes is of utmost importance to understand their complete biosynthetic pathway, which is possible via genomics or transcriptomics. Fifteen and nineteen critical enzymes have been recently identified as involved in MVA and MEP pathways in *A. heterophyllum* and *A. carmichaelii*, respectively (Pal et al., 2015; Rai et al., 2017). Another study on *A. carmichaelii* revealed the involvement of 843 unigenes in the biosynthesis of C19 -diterpenoid alkaloids, which include 22 unigenes associated with the MEP pathway and 34 unigenes related to the MVA pathway (Yang et al., 2020). Similarly, 139 A*. vilmorinianum* genes were involved in the biosynthesis of diterpenoid alkaloids, among which 3-hydroxy-3-methyl glutaryl-CoA reductase (HMGR), one of the critical enzymes in the MVA metabolic pathway, is regulated by a microRNA miR6300 (Li et al., 2021). Transcriptomics and physiological analyses of *A. kusnezoffii* suggested that plants can increase the synthesis of its defense compounds along with asexual development immediately after flowering, and these defense compounds, stored in primary roots, can be sacrificed for other processes (Tang et al., 2021).

Functional annotation of transcripts of both root and stem transcriptome in *A. heterophyllum* revealed that four genes (Hydroxymethylglutaryl-CoA reductase (HMGR), Mevalonate diphosphate decarboxylase (MVD), Mevalonate kinase (MVK), and 1-Hydroxy-2-methyl-2-(E)-butenyl 4-diphosphate synthase (HDS)) show higher transcript abundance in roots compared to shoots, suggesting their imperative increased role in secondary metabolites synthesis in roots. High expression of HMGR gene is also seen in *A. vilmorinianum* (Li et al., 2021) and *A. carmichaelii* roots (Rai et al., 2017). HMGR is a well-known regulator of the MVA pathway of isoprene genesis for phytosterols biosynthesis (Nogués et al., 2006) and is also involved in shikonin plastidial monoterpenes biosynthesis in *Arnebia euchroma* (Singh et al., 2010). In addition, genes like MVK and HDS were found responsible for producing terpenoid-indole alkaloids in *C. roseus* (Schulte et al., 2000; Ginis et al., 2012). These results provide ample evidence for the involvement of multiple MVA/MEP pathways genes in the biosynthesis of aconites in *A. heterophyllum* (Malhotra et al., 2014). Researchers have also identified a complete atisine biosynthetic pathway connecting glycolysis, MVA/MEP, serine biosynthesis, and diterpene biosynthetic pathways in *A. heterophyllum* showing 2-4 fold upregulated genes involved in glycolysis, serine biosynthesis, and diterpene biosynthesis in roots as compared with shoots (Kumar et al., 2016).

Transcription factors (TFs) play a vital role in stress response and plant development by acting temporarily and spatially on diverse target genes (Jin et al., 2014). A study on *A. heterophyllum* has revealed the involvement of 3,691 and 2,520 TFs in secondary metabolites production in root and shoot, respectively, amongst which 448 TFs were common in both, whereas 236 and 105 TFs were unique in root and shoot, respectively (Pal et al., 2015). 399 ABC transporters were identified in *A. heterophyllum*, which might be putatively involved in regulating biosynthesis and accumulating bioactive compounds. The expression of five ABC transporters was further validated by quantitative PCR analysis suggesting their role in tuberous root development (Pal et al., 2015). Simple sequence repeat (SSR) markers play an important role in selection breeding, genotyping plants, genetic diversity assessment, population structure analysis, and genetic map development. *A. heterophyllum* transcriptomics has unveiled 177,438 and 118,814 potential SSRs from 56,692 root and 32,719 shoot transcripts. Similarly, *A. carmichaelii* transcriptome has suggested the presence of 16,068 SSRs from 128,183 sequences (Rai et al., 2017). Recently the scientific community has gained a lot of interest in the functional characteristics of the CYP450s family. Several studies have observed the involvement of the CYP450s in terpene synthesis (Banerjee and Hamberger, 2017; Liao et al., 2017). Out of 124 CYP450s unigenes identified in *A. carmichaelii*, twenty-one genes are upregulated in roots suggesting their involvement in the diterpene alkaloids synthesis (Rai et al., 2017). Overall, such transcriptome-analysis-based reports will serve as practical resources for further validation investigations.

### *Aconitum* in the context of the Indian medicinal system – Ayurveda

Ayurvedic medicine employs eleven species of *Aconitum* in diverse anti-inflammatory, anti-emetic, anti-rheumatic, and anti-diarrheal activities (Tai et al., 2015). They have been suggested to treat conditions related to air and space elements in the body, also called ‘vata’ disorder, and nervous and digestive disorders when combined with other herbs. Some common *Aconitum* spp. in Ayurvedic medicine are – *A. atrox*, *A. chasmanthum, A. deinorrhizum, A. falconeri, A. ferox, A. heterophyllum, A. laciniatum, A. luridum, A. palmatum, A. spicatum* and *A. violaceum* (Tai et al., 2015). *A. heterophyllum*, among them, has a plethora of effects, including antispasmodic (abdominal discomfort), anti-inflammatory, astringent (cough, diarrhea), and antiperiodic (Tai et al., 2015; Wali et al., 2022). In Ayurveda, there are several approaches to deal with the toxicity of *Aconitum*. One of the approaches is subjecting the *Aconitum* raw material to a procedure called Shodhana, which reduces the amount of toxic metabolites. A study compared the detoxification by the Shodhana method and chemical purification method wherein it was found using toxicity studies, animal studies, and TLC that the modified Shodhana procedure was less efficient than the traditional Shodhana method of detoxification (Deore et al., 2013). Another approach is to use *Aconitum* in combination with other herbs to reduce the toxicity and improve the efficacy; for example, it is used with ginger (*Zingiber officinale*) and licorice (*Glycyrrhiza glabra*) to treat respiratory disorders (Tai et al., 2015).

## Disparities and future perspective in *Aconitum* research

Medicinal plant research requires substantial attention since its usage in traditional medicine is not supported by proper benchmarking via modern scientific research based on clinical trials and population genetics. *Aconitum* spp. are one of the most toxic medicinal plants in the world, predominantly present in mountainous meadows at 2500–4500m height ranges, and their therapeutic usage is also not adequately benchmarked. Its cultivation situation becomes much worse because of its endangerment status caused by habitat loss and excessive harvesting for export and medicinal use. Several lacunas need attention from the scientific community to properly understand their medicinal usage and help in their conservation. Foremost, there have been distinct biases in the research model organism, as *A. carmichaelii* has been studied the most, followed by *A. kusnezoffii* and *A. heterophyllum* (**Figure S1**). Another significant lacuna in *Aconitum* research is that although around 300 species have been documented, more than two-thirds remain unexplored, wherein considerable potential in pharmacology and drug discovery may be untouched. Understanding the mechanisms underlying herb-drug interactions remain a less-explored area in *Aconitum* pharmacotherapy.

Regarding the metabolites identified in *Aconitum* spp., there is an immense need to increase the metabolites testing on cell lines and animal models to confer their role in diverse pathways and understand their impact on the human body. Simultaneously, emphasis should be on understanding the role of bioactive metabolites in various combinations to find their synergistic or antagonistic properties, which would be impactful in preparing new formulations for different diseases. Understanding the basic biology of these plants is also of utmost importance; therefore, research on their floral/seed/leaf biology and breeding practices should be promoted to aid in their conservation via cultivation management.

To enhance diverse molecular biology, plant breeding, and conservation research, the genomic studies of these plants should be taken as a challenge to understand their diversity, differences, metabolic pathways, physiology, and evolution. Previous research on different medicinal plants was majorly focused on finding new therapeutic compounds; however, in the present era, the attention should also shift to investigating the structure and function of medicinal plant-associated microbiota and their physiology as the production of several phototherapeutic compounds can be critically supported with the host-associated microorganisms (Köberl et al., 2013). Therefore, along with genomics, transcriptomics, and metabolomics, understanding the structure, dynamics, and function of the associated microbiota with *Aconitum* plants will provide valuable information. Furthermore, with the continuous rise in global warming, the extinction of diverse plants and animals, especially those living at high altitudes, is also happening exponentially. Therefore, governments should also support and promote research on endangered/vulnerable/near threatened species residing in their respective countries and across borders by creating awareness on increased cultivation, lesser exploitation, managed export of medicinal products, and research funding.

## Author contributions

RK, MH, and AC wrote the first draft of the manuscript. RK, MH, and AC prepared the figures and tables. GS designed, guided, reviewed, and edited the manuscript. All authors contributed to the article and approved the submitted version.

## Supplementary material

Supplementary Figure 1 Aconitum publications over the years: Among more than 300 Aconitum spp., only a few species have been well-studied. Here, we plot the top 5 Aconitum spp. based on the number of research papers available on PubMed from 2000 to 2021, in which A. carmichaelii tops the list. Each data point represents a cumulative number of previous years. A graph was generated using an R package named Rentrez.

Supplementary File 1 Distribution of 227 unique *Aconitum* spp., and its known 289 subspecies: ploidy and endangerment status

Supplementary File 2 Classification of genus *Aconitum* into its respective taxa, i.e., subgenus, section, and series, along with their known representative members, general characteristics, secreted phytochemicals, toxicity, and usage information.
