# Supplementary figures and images for: The known, unknown, and the intriguing about members of a critically endangered traditional medicinal plant genus Aconitum
Source: Front Plant Sci. 2023 Jul 28;14:1139215. doi: 10.3389/fpls.2023.1139215 (PMC10421671; doi:10.3389/fpls.2023.1139215)

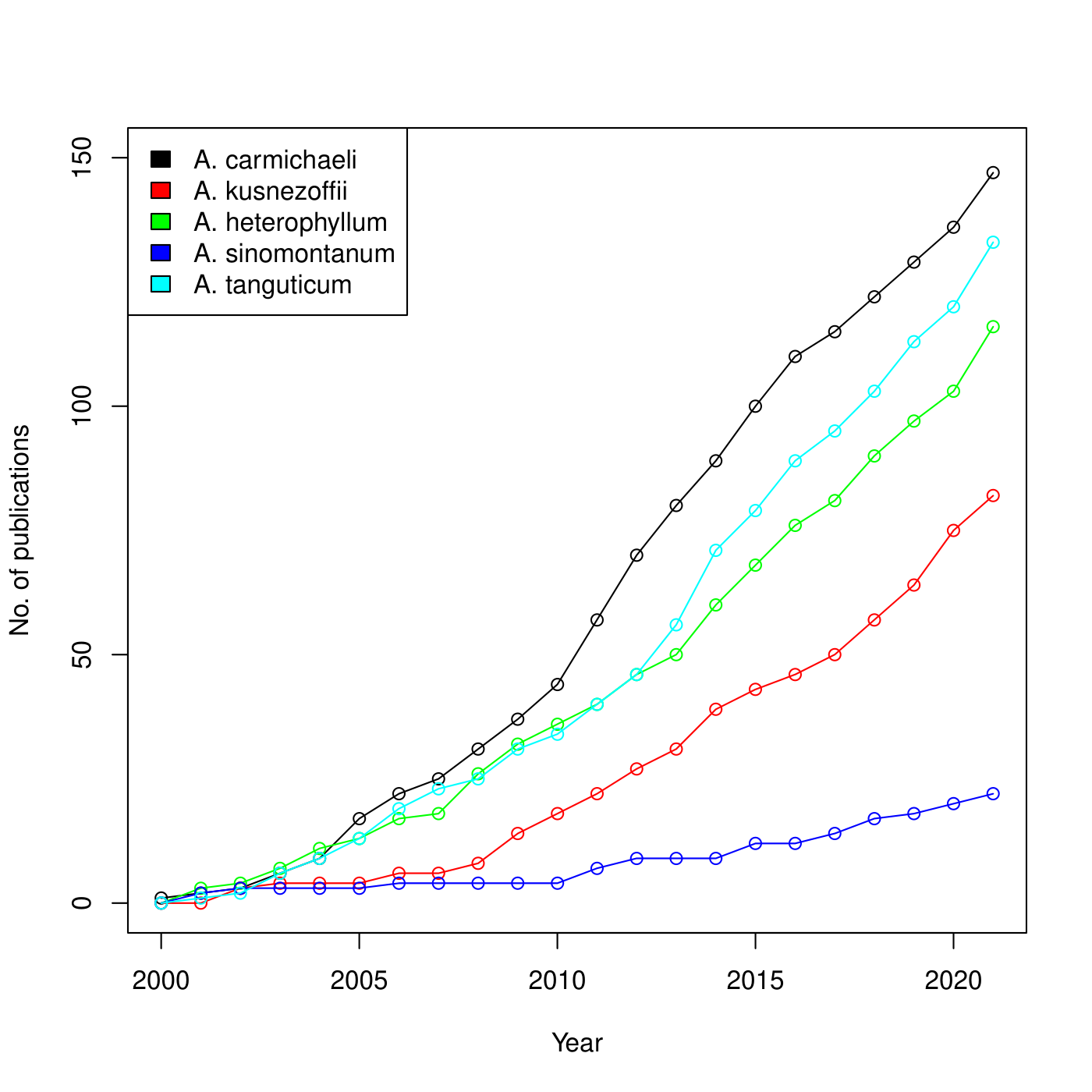

Supplement: Supplementary Figure 1 — Aconitum publications over the years: Among more than 300 Aconitum spp., only a few species have been well-studied. Here, we plot the top 5 Aconitum spp. based on the number of research papers available on PubMed from 2000 to 2021, in which A. carmichaelii tops the list. Each data point represents a cumulative number of previous years. A graph was generated using an R package named Rentrez. [file Image_1.tiff]
